# Insights into spacer acquisition of the type V-A CRISPR–Cas system of *Francisella novicida* U112

**DOI:** 10.1093/nar/gkag276

**Published:** 2026-03-30

**Authors:** Frank Hille, Chu Wang, Knut Finstermeier, Dior Beerens, Katja Schmidt, Melanie Skibbe, Emmanuelle Charpentier

**Affiliations:** Max Planck Unit for the Science of Pathogens, 10117 Berlin, Germany; Department of Microbiology and Biotechnology, Max Rubner-Institute, 24103 Kiel, Germany; Max Planck Unit for the Science of Pathogens, 10117 Berlin, Germany; Max Planck Unit for the Science of Pathogens, 10117 Berlin, Germany; Max Planck Unit for the Science of Pathogens, 10117 Berlin, Germany; Max Planck Unit for the Science of Pathogens, 10117 Berlin, Germany; Max Planck Unit for the Science of Pathogens, 10117 Berlin, Germany; Max Planck Unit for the Science of Pathogens, 10117 Berlin, Germany; Institute for Biology, Humboldt-Universität zu Berlin, 10115 Berlin, Germany

## Abstract

CRISPR–Cas systems immunize prokaryotic cells through a CRISPR adaptation process, in which short DNA fragments from foreign elements are acquired and integrated into the CRISPR array. Here, we investigated the spacer acquisition mechanism of the type V-A CRISPR–Cas system from *Francisella novicida* U112. We characterized the Cas1–Cas2 integrase *in vitro* and elucidated the sequence requirements of the pre-spacer and the CRISPR array for optimal spacer incorporation. We demonstrated that Cas2 coordinates metals at its active site to facilitate full-site spacer integration. Furthermore, we introduced this spacer acquisition system into *Escherichia coli* cells and observed that, *in vivo*, all Cas proteins are required for efficient type V-A adaptation, with Cas12 significantly improving adaptation efficiency. We showed that spacers were acquired preferentially from plasmids encoding *cas* genes, and from genomic regions of prophages and origins of replication. In addition, we found that Cas4 possesses a 3′–5′ exonuclease activity against single-stranded DNA and an ATP-independent unwinding activity towards double-stranded DNA. Cas4 interacts with the Cas1–Cas2 complex and processes pre-spacers in a PAM-dependent manner. The presence of Cas4 *in vivo* ensures that new spacers are derived from DNA immediately downstream of a 5′-TTTN-3′ PAM, which is critical for targeting invaders.

## Graphical Abstract

**Graphical Abstract:**
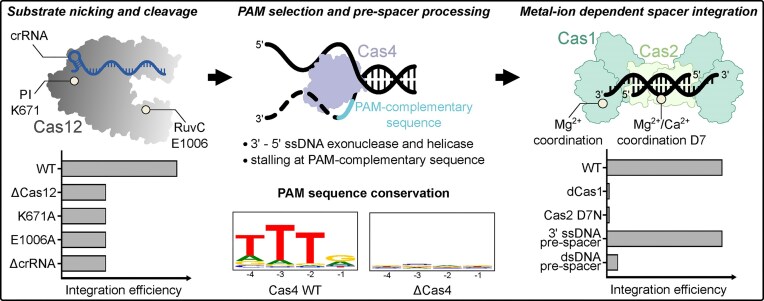

## Introduction

Prokaryotes constantly fall prey to bacteriophages and other invading mobile genetic elements (MGEs) such as plasmids or transposons [1]. To adapt to and protect themselves against such stressors, bacteria have evolved sophisticated innate and adaptive immune systems [2, 3]. Clustered Regularly Interspaced Short Palindromic Repeats and CRISPR-associated genes (CRISPR–Cas) are RNA-guided adaptive immune systems that defend bacteria and archaea against MGEs [4]. CRISPR–Cas loci typically consist of an array of short repetitive sequences (repeats) interspaced by DNA sequences (spacers) of foreign origin [5]. Generally, this array is preceded by an A-T rich leader sequence flanking a set of *cas* genes [6]. During the adaptation stage of CRISPR–Cas immunity, prokaryotes capture specific foreign DNA sequences and integrate them into their genomic CRISPR array to generate an immunological memory of invading MGEs [4]. These sequences are then transcribed as part of a long precursor pre-CRISPR RNA (pre-crRNA) molecule that is processed by either Cas proteins and/or cellular RNases to yield mature crRNAs [7]. Finally, mature crRNAs form a ribonucleoprotein effector complex with Cas nuclease(s), which monitors nucleic acids for a complementary protospacer sequence. Crucial to this interference process is the presence of a short protospacer adjacent motif (PAM) that allows the effector complex to distinguish between foreign DNA and the own genome, as the PAM sequence is not present in the host CRISPR array [8]. When a PAM is recognized and there is sufficient complementarity between the target DNA and the crRNA, the effector complex cleaves the target, resulting in the elimination of the invading genetic element [4].

CRISPR–Cas systems are currently divided into two classes based on the configuration of their effector complexes [9]. Multiple Cas proteins and a crRNA assemble to form the effector complex in class 1 systems, which are further divided into types I, III, and IV. Class 2 systems utilize a single multi-domain Cas protein bound to a crRNA or a tracrRNA:crRNA duplex, and comprise types II, V, and VI [10, 11].

The integration of new spacers into the CRISPR array during the adaptation stage enables the prokaryotic cell to become immune to the invading genetic element and is therefore a key process in CRISPR defence [12]. Cas1 and Cas2 are conserved in most CRISPR–Cas systems and are thought to be universally involved in the adaptation stage [6]. In the type I-E system of *Escherichia coli*, a complex of Cas1 and Cas2 proteins is necessary and sufficient for spacer integration. Mutations in the active site of Cas1 but not of Cas2 prevent spacer acquisition in this system. It was further shown that Cas1 is responsible for catalysing a transesterification reaction, leading to the incorporation of new spacers into the CRISPR array [13, 14]. Cas1 proteins display an intrinsic sequence specificity towards the leader-repeat boundary of the CRISPR array, thereby defining the integration site [15–17]. Spacer integration is a bipartite process that consists of a leader-proximal and leader-distal integration reaction, resulting in the ligation of both spacer strands to either end of the repeat and thus, yielding a fully integrated spacer. During this process, the size of the Cas1–Cas2 complex serves as a molecular ruler, which determines the overall spacer length and also the leader-distal integration site [14, 18, 19]. Aside from being a part of the adaptation complex, it is not clear whether Cas2 plays a more important role in some CRISPR–Cas systems. Numerous reports have described different nuclease activities for several Cas2 homologs against e.g. single-stranded (ss) DNA, double-stranded (ds) DNA and RNA [20–24]. Whether these enzymatic activities are required during CRISPR–Cas adaptation remains to be determined. Interestingly, the unique type V-C and V-D systems do not encode a Cas2 protein, and their repeat and spacer sequences are typically half the size of other types [11]. Frequent off-target integration events outside of the CRISPR locus have also been observed for spacer integration by the type V-C minimal integrase Cas1, suggesting the need for additional factors to ensure site-specific integration [25].

In addition to Cas1 and Cas2, some systems rely on additional Cas components to facilitate spacer acquisition. The *cas4* genes are conserved in many CRISPR–Cas loci of type I, type II, and type V systems [11, 26]. Cas4 is not required for spacer integration itself, but plays a critical role in the acquisition of PAM-associated pre-spacers suitable for interference [27–30]. Structural analysis revealed a stable Cas4–Cas1–Cas2 complex in which Cas4 interacts with the active sites of Cas1 [31]. Fusions of the *cas4* and *cas1* genes have been observed for several CRISPR–Cas systems [26]. A Cas4–Cas1 fusion from the type I-G system facilitates pre-spacer maturation and directional integration [32–34], suggesting that a tight interaction between Cas1 and Cas4 promotes PAM-dependent spacer integration. In addition to Cas4, Cas effector proteins have been reported to play important roles in spacer acquisition, such as the effector nuclease Cas9 in the type II-A system [35, 36], the nuclease Cas3 and the cascade protein complex in the type I-B [37, 38], type I-E [39–42], and type I-F systems [43–45].

Non-Cas host factors are sometimes also involved in spacer acquisition and empower the Cas1–Cas2 complex with additional activities. The host exonucleases DnaQ and ExoT have been found to trim pre-spacers and promote spacer integration in the correct orientation, thereby offsetting the need for Cas4 [46, 47]. In a variant of the type I-E system, Cas2 fuses with DnaQ and is responsible for processing pre-spacers prior to integration [48–50]. Some bacteria harbouring type III and VI CRISPR–Cas systems have Cas1 fused to reverse transcriptase (RT), enabling RNA protospacer integration into the CRISPR array [51–55]. In type I-E and I-F systems, the integration host factor (IHF) binds the leader sequence, causing a bend in the proximal DNA. This allows the binding of the Cas1–Cas2 complex to the first repeat, resulting in biased leader-side incorporation of the new spacer [44, 56]. The involvement of assisting host proteins in Cas1–Cas2-mediated integration has also been suggested for other type I systems, but their identity remains elusive [30, 57, 58].

The process by which new spacers are acquired in several types of CRISPR–Cas systems is well understood. However, the molecular mechanisms involved in the adaptation stage of type V CRISPR–Cas systems have only been partially described [59]. In this study, we characterized the role of each Cas component in spacer integration of a type V-A CRISPR–Cas system derived from *Francisella novicida* U112. We found that *in vitro*, only Cas1 and Cas2 are required for site-specific spacer integration. Cas1 is responsible for leader-side spacer integration in a Mg^2+^-dependent manner, and Cas2 coordinates Ca^2+^ or Mg^2+^ at its conserved D7 site to allow proper dimer structure, further facilitating Cas1 for full-site spacer integration. We analysed the pre-spacer and CRISPR array requirements in detail and found that the Cas1–Cas2 complex requires a 3′ overhang for integration and favours pre-spacer sizes between 27 and 29 bp. Our analysis also revealed that a palindromic sequence within the CRISPR array is important for site-specific integration. We further expressed various combinations of *cas* genes with a CRISPR array in *E. coli* cells and analysed the acquired spacers by high-throughput sequencing. We observed that *in vivo* spacers were mainly acquired from the plasmid carrying *cas* genes, and that Cas12 greatly promoted spacer acquisition efficiency. Finally, we established that Cas4 is a 3′-5′ exonuclease that binds to Cas1 and ensures that spacers are acquired from DNA flanked by a PAM sequence.

## Materials and methods

### Bacterial strains and plasmid construction

Bacterial strains and plasmids used in this study are listed in Supplementary Tables S1 and S2. Plasmids harbouring *cas* genes or the CRISPR array were generated by standard restriction-cloning procedures. The respective sequences were amplified from genomic DNA of *F. novicida* U112. Site-directed mutagenesis was achieved by rolling-circle polymerase chain reaction (PCR) on expression plasmids using primers that harboured the desired mutation (see Supplementary Table S3). The integrity of plasmids was verified by Sanger sequencing (Microsynth Seqlab).

### Protein expression and purification

The *cas1* and *cas4* genes were each cloned into the expression vectors pEC-A-HI-SUMO and *cas2* was cloned in pCDFDuet-2, respectively. The resulting Cas1 and Cas4 contained an N-terminal 6×His-SUMO-tag, and the resulting Cas2 contained an N-terminal 6× His-tag, allowing the proteins to be purified using immobilized metal ion affinity chromatography (IMAC). For the co-expression of Cas proteins (cas4–cas1–cas2; cas1–cas2), the native operon structure containing the respective genes was cloned into pCDFDuet-1, where *cas4* and *cas1*, respectively, were fused to an N-terminal 6× His-tag.

Expression vectors were introduced into *E. coli* Rosetta 2(DE3) pLysS by transformation and the cultures were grown with shaking at 37°C in LB medium supplemented with 30 µg/ml chloramphenicol and either 100 µg/ml carbenicillin (Cas1 and Cas4) or 50 µg/ml streptomycin (Cas2) until an OD_600 nm_ of 0.4–0.6 was reached. Protein expression was induced with 0.5 mM isopropyl-d-1-thiogalactopyranoside (IPTG), after which growth was continued with agitation at either 18°C for 16–18 h (Cas1 and Cas4) or 37°C for 4 h (Cas2). The cells were then harvested by centrifugation (15 min at 4°C, 4000 × *g*), resuspended in lysis buffer I (50 mM Tris, pH 7.5, 500 mM NaCl, 10 mM MgCl_2_) for Cas1, Cas2, and Cas protein complex, or in lysis buffer II (50 mM Tris, pH 6.8, 1 M NaCl, 10 mM MgCl_2_) for Cas4. The cells were disrupted by sonication (six cycles of 60 s pulse and 30 s pause).

Lysates were filtered (0.45 µM) and applied onto a 5-ml HiTrap Talon crude column (GE Healthcare) on an ÄKTA pure25 FPLC system (GE Healthcare). Proteins were washed with lysis buffer containing 5 mM imidazole and eluted with lysis buffer containing 350 mM imidazole using a linear gradient. After IMAC, Cas1 elution fractions were pooled and incubated with SUMO protease for 1 h at 4°C. The salt-concentration of the buffer was adjusted to 0.1 M NaCl and purified over a 1-ml HiTrap Heparin HP column (GE Healthcare) using a linear salt gradient from 0.1 to 1 M NaCl in 50 mM Tris (pH 7.5) before purification by size exclusion chromatography (SEC) on a HiLoad 16/600 Superdex 200 pg column in SEC buffer I (50 mM Tris, pH 7.5, 500 mM NaCl). For Cas2, IMAC elution fractions were pooled and subjected to SEC using a HiLoad 16/600 Superdex 200 pg column in SEC buffer II (50 mM Tris, pH 7.5, 500 mM NaCl, 10% glycerol). For Cas4, IMAC elution fractions were subjected to SUMO-tag cleavage and subsequently purified via SEC using a HiLoad 16/600 Superdex 200 pg column using SEC buffer III (50 mM Tris, pH 6.8, 500 mM NaCl, 10% glycerol). For the Cas protein complex, IMAC fractions were further purified using heparin column and SEC as described for Cas1. All purified proteins were further concentrated, snap-frozen in liquid nitrogen and stored at −80°C until use.

### DNA substrate preparation and *in vitro* spacer integration assay

Oligonucleotides used as pre-spacers were radioactively labelled at the 5′ end using [γ-P^32^] ATP and a T4 polynucleotide kinase (Thermo Fisher Scientific) according to the manufacturer’s instructions. Single-stranded substrates were annealed in 10 mM Tris (pH 7.5), 50 mM NaCl, and 1 mM ethylenediaminetetraacetic acid (EDTA) at 95°C for 5 min, followed by slow cooling to room temperature. Annealed oligos were purified using 10% native polyacrylamide gel electrophoresis (PAGE). Hairpin CRISPR DNA substrates were designed with 10 nucleotides self-complementarity, causing partial self-annealing, which served as a priming-site for a 5′ fill-in reaction using the Klenow polymerase (Thermo Fisher Scientific). The synthesized double-stranded hairpins were purified using 10% native PAGE.

Integration assays were conducted in 20 mM HEPES (pH 7.5), 25 mM KCl, 10 mM MgCl_2_, 1 mM dithiothreitol (DTT), 10% DMSO, 0.01% Tween 20 and included 1 µM Cas1, 5 µM Cas2, and 10 nM pre-spacer substrate, unless otherwise stated. The mixture was incubated for 15 min at 16°C and afterwards, the CRISPR substrate was added at a final concentration of 150 nM. The integration reaction was carried out at 30°C for 30 min and stopped with 25 mM EDTA and 2 mg/ml proteinase K for 30 min at 50°C. Next, an equal volume of 2× loading buffer (95% formamide, 30 mM EDTA) was added and the mix was incubated at 95°C for 5 min and immediately cooled on ice for at least 5 min. Samples were analysed by 8 M Urea-PAGE (8% PAA, 1× TBE) and visualized on a Typhoon FLA 9500 imaging system (GE Healthcare) after overnight exposure to a BAS storage phosphor screen. Integration band intensities were quantified with the software GelAnalyzer 2010a. The frequency of different integration products was calculated using the determined band intensities and the following formulas: all integration = (2 × top band + 2 × bottom band)/(total); leader-side = (2 × top-band)/(total); full-site = (2 × middle band)/(total). The band intensities were doubled to account for the pre-spacer strand, which was de-annealed due to the denaturing conditions in the PAGE gels.

### Sanger sequencing of *in vitro* integration products

For sequencing of *in vitro* integration products, Cas1 (1µM) and Cas2 (5 µM) were incubated with 1 µM of an 18 bp pre-spacer harbouring a 5-nt single-stranded overhang on both ends. As an integration target, we used the whole CRISPR array that was cloned into the pUC19 backbone. The resulting plasmid was added to the reaction at a final concentration of 10 nM. Possible half-site integration products were amplified by PCR (primers see Supplementary Table S3), checked on a 2% agarose gel and amplicons were further purified using a PCR-purification kit (Qiagen). Purified PCR products were cloned into the pCRII vector using the Dual promoter TA-cloning Kit (Thermo Fisher Scientific). The resulting plasmid was introduced by transformation into *E. coli* INVαF’. After incubation at 37°C o/n on an LB agar plate supplemented with respective antibiotics, 13 colonies per transformation reaction were picked and grown o/n at 37°C. Plasmids were then extracted (Qiagen Miniprep Kit) from the selected clones and analysed by Sanger sequencing (Microsynth Seqlab).

### Microscale thermophoresis

Binding studies were done by microscale thermophoresis (MST) on a Monolith NT.115 (NanoTemper Technologies). For this, the target protein was either covalently labelled with the RED-NHS fluorescent dye (MO-L001, NanoTemper) or non-covalently using the RED-tris-NTA dye (MO-L008), according to the manufacturer’s instructions. A constant target protein concentration of 25–50 nM diluted in assay buffer (20 mM HEPES, pH 7.5, 25 mM KCl, 0.01% Tween 20) was used. To this, a serial dilution of ligand dissolved in assay buffer was added. After short incubation, samples were loaded into MST Premium Coated Capillaries (MO-K025). Measurements were carried out with medium MST-power and 20%–40% excitation powers. The results were evaluated using either 1.5–5 s MST on-time or based on a ligand-induced fluorescence change (as indicated in the respective figures). All binding curves and dissociation constants (*K*d) were calculated using the MO. Affinity Analysis Software and GraphPad Prism.

### Cas4 enzymatic assay

Cas4 (5 µM) was incubated with 0.1 µM DNA substrates for 30 min at 18°C in 20 mM HEPES (pH 7.5), 25 mM KCl, 10 mM MgCl2, 1 mM DTT, 10% DMSO, 0.01% Tween 20. Reactions were stopped with 1 µl 250 mM EDTA and 1 µl 20 mg/ml proteinase K and incubated at 50°C for at least 30 min before an equal volume of 2× formamide buffer was added. Samples were heated to 95°C for 5 min, cooled on ice and 10 µl were loaded onto a denaturing PAA gel (8 M Urea; 15% PAA, 1× TBE). Labelled DNA substrates were visualized using a Typhoon FLA 9500 imaging system.

### *In vivo* spacer acquisition assay

To establish a heterologous system in *E. coli* BL21(DE3), the corresponding type V-A *cas* genes were cloned into a pCDFDuet-2 vector (*cas12a* in MCS-I and *cas4-Cas1–Cas2* in MCS-II) in different combinations termed pCas. The associated CRISPR array in a truncated version containing a leader-repeat-spacer1-repeat structure was constructed into a pACYC184 vector, yielding the pCRISPR plasmid (Supplementary Table S2). Transcription of both the *cas* genes and the CRISPR array was driven by a T7 promoter. The spacer acquisition assay was conducted as follows. Single colonies of *E. coli* BL21(DE3) transformed with pCRISPR and pCas were picked and grown with shaking overnight in the presence of 1% glucose and selective antibiotics (50 µg/ml streptomycin for pCas, 30 µg/ml chloramphenicol for pCRISPR) at 37°C. Overnight cultures were diluted 1:100 in 10 ml LB medium, supplemented with appropriate antibiotics and grown at 37°C with shaking to an OD_600 nm_ of 0.5. The culture was pelleted and washed with fresh media containing either antibiotic selection for pCas and pCRISPR or for pCRISPR only. Gene expression was induced with 0.5 mM IPTG and growth continued with agitation at 37°C for 20 h. Similar growth conditions were applied to strains transformed with pCas, pCRISPR, and pFliC (100 µg/ml carbenicillin for pFliC). Transcription of the *fliC* gene was controlled by a tetracycline-dependent promoter. The subculture was pelleted and washed with fresh media containing antibiotic selection for pCRISPR at OD_600 nm_ 0.5. Gene expression was induced by 0.5 mM IPTG with or without 200 ng/ml anhydrotetracycline, after which growth was continued with agitation at 37°C for 20 h. Cells were collected and normalized to an OD_600 nm_ of 1.0 in 200 µl, pelleted, and resuspended in 100 µl ddH2O before storage at −20°C for further PCR reaction. Similar approaches were repeated in BL21-AI cells with the addition of 2 g/l *L*-arabinose and 0.5 mM IPTG for gene expression. To detect acquired spacers, a 20 µl PCR reaction was set up using 2 µl of normalized cell suspension, a forward primer (OLEC3771) that binds to the leader sequence and a reverse primer (OLEC6679) that binds to the existing spacer in pCRISPR. PCR products were separated on a 2.5% agarose gel for 90 min at 90V, gels were stained with SYBR gold or ethidium bromide and visualized using a Typhoon FLA 9500 imaging system.

### High-throughput sequencing of acquired spacers *in vivo*

To prepare the library for next generation sequencing (NGS), a 50 µl barcoding PCR reaction was setup using 5 µl normalized cell suspension. A reverse primer (OLEC14485) annealing to the existing spacer in pCRISPR was used to amplify the target area for 1 cycle. The primer also contains a linker sequence and an N10 Unique Molecular Identifier (UMI) to tag individual DNA molecules (see Supplementary Fig. S6A). The PCR product was purified using AMPure XP Beads (Beckman Coulter Life Sciences) with a 1.2× bead ratio. Afterwards, a forward primer (OLEC14483) binding to the leader sequence and a reverse primer (OLEC14484) binding to the linker sequence was used to amplify the UMI-tagged DNA amplicons for 25 cycles, followed by a 1.4× AMPure XP Bead purification. The libraries were pooled after barcoding using PCR primers with standard i7 index sequences, purified with 1.2× AMPure XP beads and further separated on an 8% native polyacrylamide gel. The gel sections indicating the presence of acquired additional nucleotides were excised and purified.

Following checking of library concentration using Qubit and quality control using Agilent’s Bioanalyzer, the sequencing was carried out at the Sequencing Service Facility of the Max Planck Institute for Molecular Genetics on Illumina’s MiSeq system V3 with a PE300 protocol, or on the NextSeq2000 P1 system with a PE150 protocol yielding between 80 000 and 1 700 000 clusters per sample with a median of 650 000 clusters. Sequencing data are available at the European Nucleotide Archive (accession: PRJEB87316). Reads were processed by identification of the 10 bp UMI followed by the identification of the newly acquired spacers in the forward and reverse reads, separately utilizing the spacer array repeat sequence before and after each spacer sequence and allowing for up to two mismatches in the repeat sequence. For each unique spacer sequence and sample, the unique number of UMIs was considered the true number of original molecules.

Identified spacers were mapped to the sample-specific circularized genome and plasmid reference sequences using BWA Version 0.7.18-r1243-dirty with the following mapping parameters diverging from the defaults: -k 4 -w 7 -B 1 -O 1 -T 22 to accommodate the short nature of the spacers [60] as well as Samtools V 1.20-17-g078b220 [61]. Sequence duplicates caused by the circularization of the references were removed. Resulting positions were extracted with the Python library pysam V0.21.0. A maximum of two mismatches including insertions and deletions between spacer and reference sequence were allowed. Clustering was performed to compensate for PCR and sequencing errors. All spacers with identical mapping coordinates were assigned the most frequent spacer sequence identified. Information about annotated affected genes (either strand) was added to the resulting tables. Visualizations of the distribution of spacer origins were created using custom Python scripts with MatPlotLib library V3.7.1.

## Results

### Site-specific spacer integration *in vitro* requires only Cas1 and Cas2

**Figure 1.**
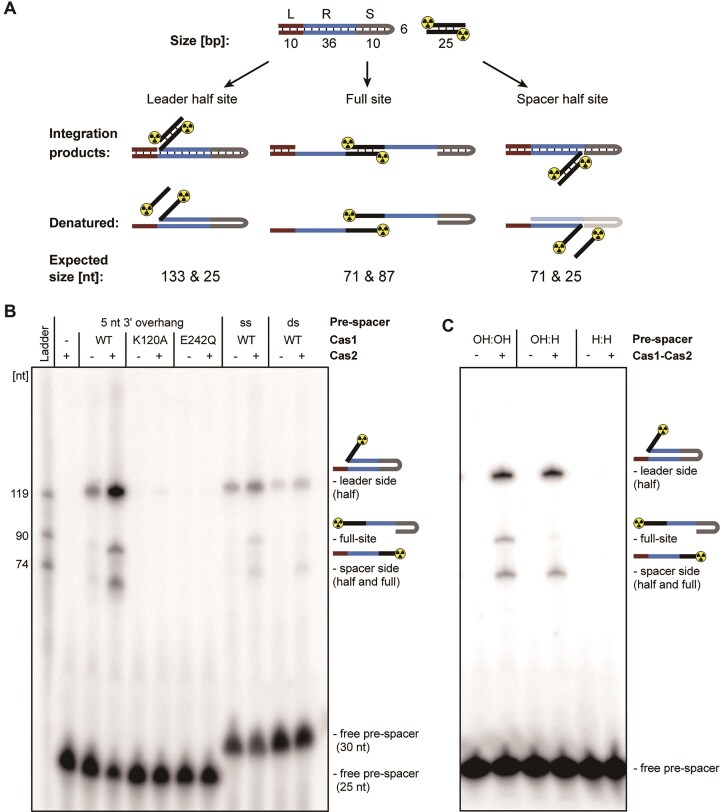
Full-site spacer integration requires Cas1 and Cas2. (**A**) Schematic hairpin CRISPR DNA integration assay (adapted from Wright and Doudna). The hairpin CRISPR DNA substrate resembling the CRISPR array consists of a leader (L), a repeat (R), and a spacer (S) portion. Possible integration products and their respective sizes are shown. The pre-spacer was radiolabelled at the 5′ end (indicated by radiation symbol). Denaturing conditions lead to specific DNA sizes depending on the nature of the integration products. (**B**) *In vitro* integration assay by addition of Cas2 WT, Cas1 WT, and two Cas1 active site mutants (K120A and E242Q). The integration assay was conducted using three different pre-spacers [double-stranded (ds), single-stranded (ss), and partially single-stranded (5 nt 3′ overhang)]. Shown is a representative gel of three replicates. (**C**) Dideoxynucleotides (ddNTPs) at the 3′ end (H) of the pre-spacer are not integrated by Cas1–Cas2. Pre-spacers harbouring a ddNTP on both strands (H:H) or on one strand (OH:H) were used. The regular deoxynucleotide double strand is indicated with OH:OH. Shown is a representative gel of three replicates.

The *F. novicida* type V-A CRISPR–Cas system comprises the genes *cas12a* (formerly *cpf1*), *cas4, cas1, cas2*, and a CRISPR array containing 9 spacers (Supplementary Fig. S1A). To analyse the mechanism of spacer integration, we overexpressed and purified the respective Cas1 and Cas2 proteins and studied their capacity to integrate spacer sequences into a CRISPR array. The *in vitro* integration assay was adapted from an assay by Wright and Doudna [17], which allows the discrimination of half- and full-site integration products using a hairpin CRISPR DNA substrate and radiolabelled pre-spacers (Fig. 1A).

We observed that Cas1 alone was able to generate half-site spacer integration products at the leader end of the repeat (Fig. 1B, lane 3). Adding Cas2 to the reaction increased leader-side specific integration and facilitated full-site integration (Fig. 1B, lane 4). Amino acid changes in the active site of Cas1 (K120A and E242Q) almost abolished integration, confirming that Cas1 is responsible for catalysis of the integration reaction (Fig. 1B, lanes 5–8). The results of this assay demonstrate that the type V-A Cas1–Cas2 integrase functions similarly to the previously described Cas1–Cas2 complex of type II-A systems [16, 17, 62], and does not require additional factors for integration [56, 63, 64].

To confirm that the observed bands corresponded to the expected integration products, we used pre-spacers with one or both strands labelled with a ddNTP at the 3′ end, which prevents the nucleophilic attack of the 3′ OH of the pre-spacer and thus DNA integration. No integration bands were detected when both 3′ ends of the pre-spacer were labelled with a ddNTP (Fig. 1C). Labelling one strand led to the generation of only half-site integration products, confirming the functionality of our assay. Moreover, we observed that there was a clear reduction in integration efficiency when the pre-spacer was completely double-stranded compared to a pre-spacer that was double-stranded, but harboured a single-stranded overhang at the 3′ end (Fig. 1B). These results further indicate that pre-spacers must be sufficiently processed to allow efficient spacer integration.

### A conserved aspartate residue in Cas2 is necessary for full-site spacer integration and is involved in binding of divalent metal ions

**Figure 2.**
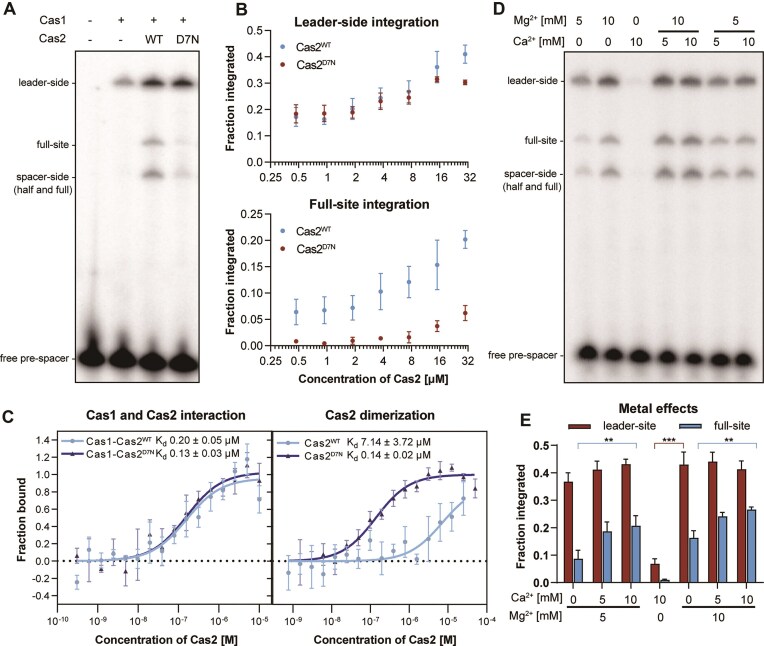
Full-site integration efficiency is affected by Ca^2+^ and a conserved aspartate residue of Cas2. (**A**) *In vitro* integration assay using Cas1 WT and Cas2 WT or Cas2 D7N. The hairpin CRISPR DNA substrate was used to distinguish half- and full-site integration products. Shown is a representative gel of three replicates. (**B**) Quantification of *in vitro* leader-side and full-site integration products. The integration assay was carried out using 1 µM Cas1 and varying concentrations of either Cas2 WT or Cas2 D7N. Error bars indicate the standard deviation of three replicates. (**C**) MST analysis of Cas1–Cas2 interaction and Cas2 dimerization. For Cas1 and Cas2 interaction, RED-NHS labelled Cas1 was incubated with serial dilutions of Cas2 WT or Cas2 D7N. For Cas2 dimerization, RED-NHS labelled Cas2 WT or Cas2 D7N was incubated with serial dilutions of unlabelled Cas2 or Cas2 D7N. Binding was measured at 23°C and dose response curves were calculated based on the ligand-induced fluorescence change. Error bars indicate the standard deviation of three replicates. (**D, E**) Effects of Ca^2+^ and Mg^2+^ on leader-side and full-site integration efficiencies of Cas1 and Cas2 using a 28 bp pre-spacer substrate containing single-stranded 3′ ends. Shown is one representative gel image and error bars indicate quantification of three replicates. Statistical significance was analysed using two-way ANOVA with Tukey’s multiple comparison test. Asterisks indicate significant changes in integration efficiency. *P-*values were reported in accordance with APA style: *P *< .033 (*), *P *< .002 (**), *P *< .001 (***).

Our data suggest that Cas2 is essential for full-site spacer integration and enhances the intrinsic capacity of Cas1 for leader-side integration. To investigate the role of Cas2 during spacer acquisition, we altered a highly conserved aspartate residue at the N-terminus of Cas2 (D7N, Supplementary Fig. S2A). We observed that in the presence of Cas1, Cas2 (D7N) still promoted the generation of the leader half-site integration product, whereas full-site integration was nearly abolished (Fig. 2A). Similar experiments with the type I-E Cas1–Cas2 integrase of *E. coli* showed that amino acid substitutions in the active site of Cas2 (E9A and E9Q) had no effect on spacer integration *in vivo* or *in vitro* [13, 14]. Only Cas2 amino acid substitutions involved in the interaction with Cas1 abolished spacer acquisition in type I-E CRISPR system [13, 14].

To further understand the influence of Cas2, we conducted the spacer integration experiments with varying concentrations of Cas2 WT or D7N, while keeping the Cas1 concentration constant. For Cas2 WT, we observed that increasing ratios of Cas2 to Cas1 favoured the generation of leader half- and full-site integration products (Fig. 2B), which implies that the Cas1–Cas2 interaction might be rather transient. Thus, an excess of Cas2 could shift the binding equilibrium of Cas1 and Cas2 towards an integration complex. Titration of the Cas2 (D7N) variant resulted in identical frequencies of leader half-site integration compared to WT, whereas only a slight stimulation of full-site integration was observed at excessive Cas2 (D7N) concentrations (Fig. 2B).

To exclude the possibility that the Cas2 (D7N) variant affects the interaction with Cas1, we examined Cas1–Cas2 complex formation by MST. We fluorescently labelled Cas1 and titrated Cas2 WT or the D7N mutant (Fig. 2C). No significant difference was detected between the affinities of Cas1 bound to Cas2 WT or Cas2 D7N (*K*_d_^WT^ 0.20 ± 0.05 µM and *K*_d_^D7N^ 0.13 ± 0.03 µM), suggesting that the aspartate substitution does not affect the interaction of Cas2 with Cas1. Since Cas2 normally forms dimers within the CRISPR adaptation complex [13, 62], we further investigated the dimerization affinity of Cas2 WT or Cas2 D7N. Interestingly, we observed a much stronger (*P *< 0.01) interaction of Cas2 mutant dimerization compared to WT (*K*_d_^WT^ 7.14 ± 3.72 µM and *K*_d_^D7N^ 0.14 ± 0.02 µM) (Fig. 2C), indicating that the aspartate residue is relevant for proper Cas2 dimerization.

Previous studies have observed that the N-terminal aspartate residue of Cas2 is involved in Mg^2+^ coordination and pre-spacer contact [62]. Therefore, we used MST to evaluate the metal coordination of Cas2. Surprisingly, Cas2 binds only weakly to Mg^2+^ (*K*_d_ 1.92 mM) but has a stronger preference for Ca^2+^ coordination (*K*_d_ 0.42 mM) (Supplementary Fig. S3A and B). We further investigated the effect of Ca^2+^ on spacer integration *in vitro*. The addition of Ca^2+^ alone did not significantly promote the generation of integration products (Fig. 2D and E), indicating that the active site of Cas1 is strictly dependent on Mg^2+^ as a cofactor for spacer integration. The addition of both Mg^2+^ and Ca^2+^ increased the amount of full-site integration products two-fold compared to the presence of Mg^2+^ alone (Fig. 2D and E). Considering that Cas1 cannot utilize Ca^2+^ ions for spacer integration, our results indicate that the interaction of Ca^2+^ with Cas2 may be responsible for the increased integration frequencies. Indeed, we found that the binding affinity of Cas2 D7N to Ca^2+^ (*K*_d_ 4.61 mM) was significantly decreased compared to the WT protein, whereas the binding constant of Cas2 D7N to Mg^2+^ ions was barely altered (*K*_d_ 1.91 mM) (Supplementary Fig. S3A and B). We further checked the impacts of the Cas2 D7N and metal ion coordination on Cas2 dimerization (Supplementary Fig. S3C). We observed that the presence of Ca^2+^ or Mg^2+^ significantly promoted the dimerization of Cas2, whereas this effect was not significant for Cas2 D7N, which was already tightly dimerized (Fig. 2C and Supplementary Fig. S3C) and might have difficulties to coordinate the Ca^2+^. An AlphaFold structure prediction [65] supported our observations by showing that the D7 sites of the Cas2 dimer could form a negatively charged channel coordinating two Ca^2+^ ions in an electrostatic potential map (Supplementary Fig. S2B). This metal coordination would potentially support a flexible interaction with the Cas2 dimer and might guide Cas1 to the second integration site.

### The pre-spacer and CRISPR array require specific features for efficient full-site integration

**Figure 3.**
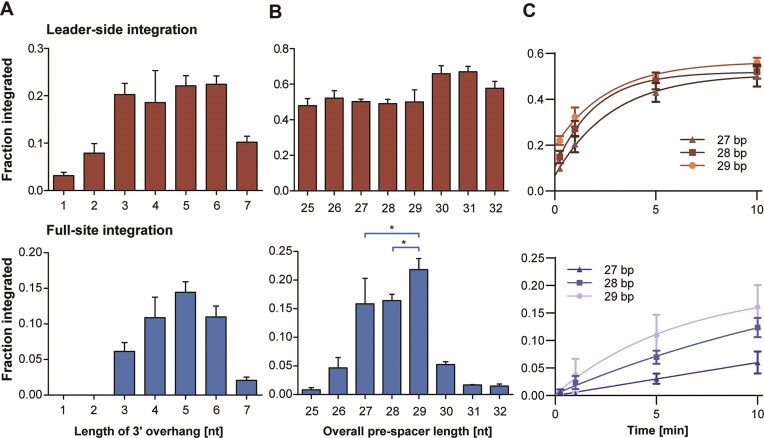
Cas1–Cas2 prefers specific pre-spacer substrates for integration. (**A**) Quantification of leader-side (half-site) and full-site integration events for pre-spacers with differently sized 3′ overhang lengths (overall pre-spacer length was 28 bp). (**B**) Quantification of leader-side and full-site integration events for pre-spacers with different lengths (pre-spacers contained a 5 nt 3′ single-stranded overhang). Statistical significance was analysed between pre-spacer length 27 versus 29 and 28 versus 29 nucleotides, respectively, using two-way ANOVA with Tukey’s multiple comparison test. Asterisks indicate significant changes in integration efficiency, *P-*values were reported in accordance with APA style: *P *< .033 (*), *P *< .002 (**), *P *< .001 (***). (**C**) Integration kinetics for 27–29 nt pre-spacers (time points 0.25, 1, 5, 10 min). Shown are quantifications of leader-side events and full-site integration products, nonlinear least squares fit using the GraphPad Prism one-phase association. Error bars indicate the standard deviation of three replicates.

We previously found that the Cas1–Cas2 complex prefers double-stranded pre-spacers with a single-stranded 3′ overhang for efficient integration (Fig. 1). To further investigate how Cas1–Cas2 selects certain DNA substrates for integration, we varied the length of the 3′ overhang and the overall size of the pre-spacer, respectively, and compared their integration efficiencies (Fig. 3). We observed that both half-site and especially full-site integration was strongly dependent on the presence of a single-stranded 3′ end. No full-site integration was observed when the overhang was <3 nucleotides, whereas a 4–6 nucleotide overhang is the optimal length for full-site integration (Fig. 3A).

To evaluate the integration efficiency of different sized pre-spacers, we kept the length of the 3′ overhang constant at 5 nucleotides in the tested DNA substrates, and expanded or reduced the double-stranded region. Cas1 and Cas2 appeared to be quite flexible in accepting pre-spacers of different sizes for leader half-site integration, as long as they contained a single-stranded 3′ overhang (Fig. 3B, upper panel). For full-site integration, however, the pre-spacer sizes with the highest integration efficiencies were restricted to a length between 27 and 29 nucleotides (Fig. 3B, lower panel), which corresponds to the spacer sizes found in the native CRISPR array of the analysed *F. novicida* strains (Supplementary Fig. S1B). The majority of half-site integration events occurred at the leader-side, suggesting that the first integration reaction takes place at the leader-end (Fig. 3B). The half-site product is subsequently converted to a fully integrated spacer but only if the pre-spacer has the appropriate length. We further determined their integration kinetics to rule out any discrepancies between the integration efficiencies of the 27–29 nucleotide pre-spacers. While no pronounced difference between the 28 and 29 nucleotide long substrates was observed, the 27-nucleotide substrate showed the lowest integration rate (Fig. 3C).

**Figure 4.**
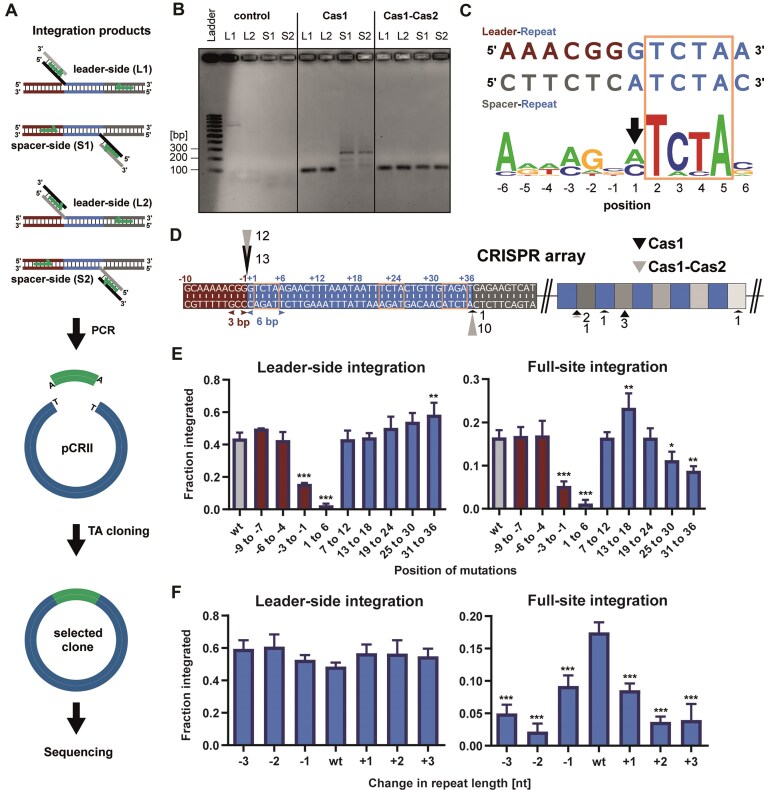
CRISPR array sequence requirements for *in vitro* integration. (**A**) Possible half-site integration products were amplified using primers indicated by green arrows. The resulting amplicons were integrated into the pCR™II vector using a TOPO™ TA cloning approach. The plasmids were subsequently introduced into *E. coli* INVαF' by transformation and analysed by Sanger sequencing. (**B**) Gel electrophoresis analysis on a 2% agarose gel of PCR products from the *in vitro* integration assay. The integration assay was carried out using an 18 bp pre-spacer substrate containing 5 nt single-stranded 3′ overhangs at both ends and a plasmid harbouring the complete type V-A CRISPR array. (**C**) WebLogo alignment of the Cas1 integration sites determined by sequencing of the spacer-side integration products [see panel (D)]. Six base pairs up- and downstream of the integration sites were used for alignment (*n* = 7; excluding the proper integration product at the first repeat-spacer boundary). A black arrow indicates the integration site. The palindromic region of the repeat is indicated by an orange rectangle. (**D**) Sequence of the type V-A CRISPR array showing the first repeat with 10 nucleotides of the leader (red) and 10 nucleotides of the first spacer (grey). Pre-spacer integration positions were determined by Sanger sequencing. Black and grey triangles indicate pre-spacer integration sites for Cas1 and Cas1–Cas2, respectively, on the plus (leader-side) and minus (spacer-side) strands of the CRISPR array. For each integration assay, 13 clones were analysed by Sanger sequencing. Shown are the results of sequenced pCRII plasmids selected because they carried an insert after TA cloning. Palindromic regions of the repeat are indicated by orange rectangles. (**E**) Quantification of leader-side and full-site integration products on mutated CRISPR arrays. The leader and the repeat were mutated in intervals of 3 and 6 bp, respectively [indicated by double headed arrows in panel (D)]. See also Supplementary Table S3 for sequences alteration details. (**F**) Quantification of leader-side and full-site integration products on CRISPR arrays harbouring base insertions (+1,2,3) and deletions (−1,2,3) in the middle of the repeat. Error bars indicate the standard deviation of three replicates. Statistical significance was analysed using one-way ANOVA with Dunnett’s multiple comparison. Asterisks indicate significant differences compared to wt, *P*-values were reported in accordance with APA style: *P *< .033 (*), *P *< .002 (**), *P *< .001 (***). Representative gels used for quantification for panels (E) and (D) are shown in Supplementary Fig. S4.

Next, we wanted to analyse which sequence motifs of the CRISPR array are necessary for pre-spacer recognition and incorporation by Cas1–Cas2. We therefore performed an *in vitro* integration assay using a plasmid harbouring the complete CRISPR locus from *F. novicida* U112 as an integration target. Possible integration products were amplified by PCR, cloned into a pCRII vector and introduced into *E. coli* by transformation. Single colonies were selected and their plasmids were analysed by Sanger sequencing (Fig. 4A). Gel electrophoresis analysis of the PCR products obtained after integration showed a distinct band for leader-side integration when Cas1 or Cas1–Cas2 were present in the integration reaction (Fig. 4B). Amplification of spacer-side products yielded a single band when Cas1 and Cas2 were present, but multiple bands when only Cas1 was present, indicating integration events at different positions in the CRISPR plasmid. Sequence analysis of the integration products demonstrated that even in the absence of Cas2, Cas1 was able to specifically integrate spacers at the leader-repeat boundary (Fig. 4D). Further sequence analysis of spacer-side amplicons confirmed multiple integration sites within the CRISPR array in the presence of Cas1 alone (Fig. 4D). Aligning the sequences of the non-canonical integration sites revealed a conserved 4-nucleotide motif, which was identical to the palindromic sequences present on both ends of the repeats, thus indicating that this short motif is essential for target recognition by Cas1 (Fig. 4C).

We observed that the presence of Cas2 in the integration reactions shifted the preference of the spacer-side integration position towards the end of the first repeat (Fig. 4D). These data strongly suggest that Cas1 is responsible for leader-side integration by recognizing the repeat palindrome, while Cas2 guides Cas1 to the proper second integration position at the spacer-side.

To gain a better understanding of the requirements of the CRISPR array for site-specific integration, we altered the sequence of the leader and the repeat by substituting, deleting or adding 1–6 nucleotides at the indicated positions and then calculated half- and full-site integration efficiencies using radiolabelled pre-spacer and hairpin CRISPR DNA (Fig. 4D and E, Supplementary Fig. S4, and Supplementary Table S3).

Using this approach, we were able to confirm the importance of the palindromic sequence within the CRISPR array for site-specific integration. The type V-A repeat harbours two short palindromic motifs: the sequence TCTA is present at both ends of the repeat and at positions 22–25, whereas the motif AAAT is located in the middle of the repeat at positions 14–17 and 19–22 (Fig. 4D). Mutations in the leader sequence proximal to the repeat reduced integration efficiency by more than half compared to the wt sequence, indicating that these three base pairs are required for integration (Fig. 4E, −3 to −1). Mutations in the first six bases of the repeat drastically reduced half- and full-site integration efficiencies (Fig. 4E, 1 to 6). Further downstream mutations in the repeat did not alter the frequency of leader half-site products (Fig. 4E). Interestingly, mutations in the 3′ end of the repeat affected full-site integration, but not to a degree that was observed with the Cas1–Cas2 integrase of the type II-A system, where mutations in this area completely abolished full-site integration [17]. Mutations in the repeat sequence at positions 25–30 showed a slight decrease in full-site integration events, whereas mutations in the middle of the repeat (positions 13–18) improved full-site integration (Fig. 4E). The base substitutions we used (A to T and C to G) may have favoured binding to the repeat by the Cas1–Cas2 complex. It is possible that purine and pyrimidine substitutions (A to G and T to C, respectively) would result in different spacer incorporation efficiencies. Sequence alterations in the downstream portion of the repeat led to the accumulation of leader half-site integration products.

This suggests a model whereby spacer integration is a bipartite reaction starting with a leader half-site product, which is then converted into a full-site product by ligation of the second pre-spacer end to the downstream end of the repeat. In case the integrase is incapable of full-site integration, half-site intermediates accumulate. Changes in repeat length drastically decreased the frequency of full-site integration events (Fig. 4F). Therefore, it appears that in type V-A adaptation, the second integration event relies on both sequence conservation in the spacer proximal repeat end and a ruler mechanism.

### Cas12 greatly enhances the spacer acquisition efficiency from mobile genetic elements *in vivo*

We were able to characterize Cas1 and Cas2 as key players in the type V-A spacer acquisition process, as both proteins are crucial for spacer integration *in vitro*. To gain a deeper insight into the type V-A adaptation process and to understand the role of Cas12 and Cas4, we studied spacer integration *in vivo*.

**Figure 5.**
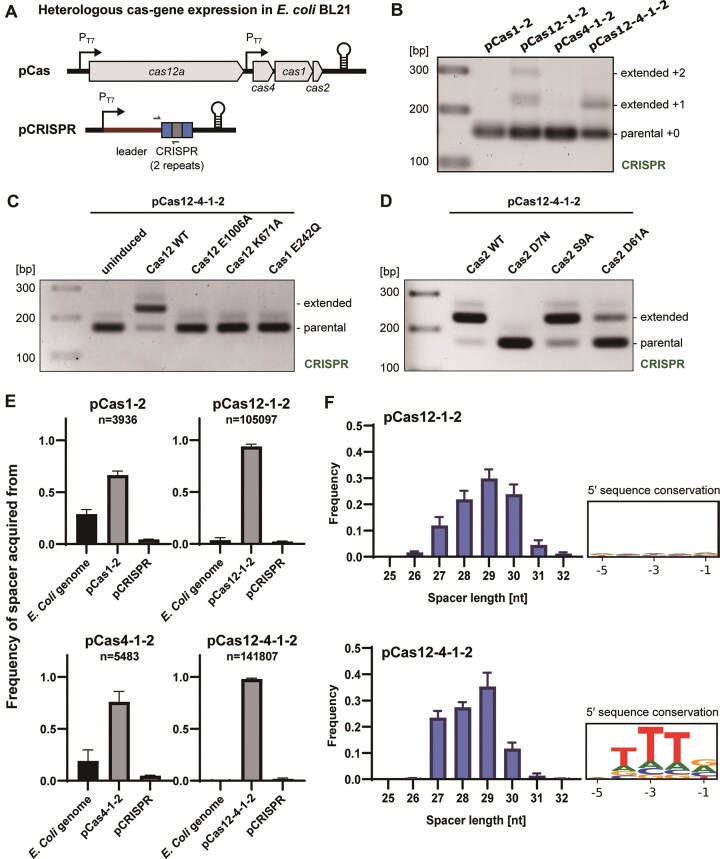
Cas12 significantly enhances spacer acquisition efficiency *in vivo*. (**A**) Experimental setup to study heterologous *in vivo* spacer acquisition in *E. coli* BL21. Two plasmids carrying the *F. novicida* type V-A *cas* genes (pCas) and a truncated CRISPR array (pCRISPR), respectively, were introduced into *E. coli* BL21 by transformation. (**B–D**) Agarose gel showing amplified CRISPR arrays following *in vivo* integration assay using the wt *cas* genes and various mutants with antibiotic selection for pCRISPR upon gene expression. The combinations of Cas proteins are noted above. Shown are representative gels of three replicates. (**E**) Relative frequencies of spacers acquired from pCas, pCRISPR, or *E. coli* genome following NGS analysis of the amplified CRISPR array after *in vivo* acquisition assay. The frequencies were calculated from the absolute count of distinct spacers acquired from each origin, identified using UMIs, and then normalized by the total number of spacers. Error bars indicate the standard deviation of three replicates. The total numbers of acquired spacers for each tested strain are indicated. (**F**) Length distribution of acquired spacers based on NGS sequencing data. Error bars indicate the standard deviation of three replicates. Logo plots of conserved sequences directly upstream of protospacers acquired *in vivo* in both tested strains are indicated in black boxes (positions −5 to −1 referring to protospacer location).

We established a two-plasmid acquisition assay in the heterologous host *E. coli* BL21, where the first plasmid (pCas) harbours the type V-A *cas* genes in different combinations under the control of a T7 promoter, and the second plasmid harbours a truncated version of the *F. novicida* U112 CRISPR array consisting of a leader-repeat–spacer-repeat sequence (pCRISPR) (Fig. 5A). The annotation of the *F. novicida* U112 genome in NCBI (accession: NZ_CP009633) indicates that the coding sequence (CDS) of *cas4* starts with the non-canonical start codon TTG, which is 21 amino acids shorter compared to closely related *cas4* genes. To investigate the properties of Cas4, we reconstituted the *cas4* gene during cloning by correcting the nonsense mutation (TAG stop codon→ TTG leucine at position 6) and refer to this version as the ‘wild type’ in this study (Supplementary Fig. S1A).

Bacteria were grown to mid-log phase when expression of the *cas* genes and the CRISPR array was induced in the presence of the antibiotic selecting only for pCRISPR. Cells were harvested 20 h after induction and the region containing the newly acquired spacers was amplified by PCR and finally visualized on an agarose gel (Fig. 5B–D and Supplementary Fig. S5). While adaptation requires only Cas1 and Cas2 *in vitro*, we barely detected spacer acquisition *in vivo* when only Cas1-2 or Cas4-1-2 were expressed from pCas for 20 h (Fig. 5B). We continued the culture for up to 11 days and detected only faint extended spacer bands after days of induction (Supplementary Fig. S5A), indicating a very limited spacer acquisition efficiency. Interestingly, we observed a much higher spacer acquisition efficiency when Cas12 was present (Fig. 5B), as extended bands corresponding to one or more new spacers could be observed after gel electrophoresis of the PCR products. Remarkably, amino acid substitutions of the active sites of Cas12 (E1006A or K671A), Cas1 (E242Q), or Cas2 (D7N) did not yield any detectable integration products *in vivo* (Fig. 5C and D), further demonstrating the involvement of Cas12 and Cas1–Cas2 in type V-A adaptation and confirming our *in vitro* observations regarding the importance of the active site of Cas2. Substitutions at the other conserved sites of Cas2 (S9A and D61A, Supplementary Fig. S2) did not abolish spacer integration (Fig. 5D). Moreover, we did not detect any extension of the CRISPR array when crRNA production was prevented (Supplementary Fig. S5C), indicating that the apoenzyme Cas12 is not sufficient to promote spacer integration, but requires bound crRNA to activate the enzyme.

We went on to analyse the origins of the acquired spacers by tagging the spacer acquisition sites with UMIs to alleviate the PCR duplication problem, then excising the bands corresponding to the extended CRISPR array and performing NGS (Supplementary Fig. S6A). The identified spacers were then mapped to the plasmids and the *E. coli* genome. The regions shared between plasmids and genome, e.g. *lacI* gene site, were excluded from the mapping analysis. The regions common in a single source were averaged by spot number. Spacer acquisitions were detected in all tested strains after deep sequencing (Fig. 5E), indicating that Cas12 is not strictly essential for naïve adaptation. However, in the presence of Cas12, we detected much more spacers and the majority of these spacers (~90%) were mapped to the pCas plasmid (Fig. 5E). In comparison, when the assays lacked Cas12 or included antibiotic selection for pCas, we found an increased proportion of acquired spacers derived from the *E. coli* genome (Fig. 5E and Supplementary Fig. S5D and S6B). Similar observations have been reported in type I-E and I-F systems, where the origin of new spacers showed strong preference for the plasmid carrying Cas1 and Cas2 [66, 43, 45]. To further understand the mechanism, we introduced another high copy number plasmid with or without induction of the flagellin gene *fliC*, which encodes a highly expressed bacterial protein and is biochemically stable in *E. coli* [67]. Interestingly, we found that this high copy number plasmid had little effect on the high rate of acquisition from pCas, regardless of whether *fliC* was overexpressed (Supplementary Fig. S6C). This suggests that the origin of spacer acquisition is not random and that pCas is an efficient target for spacer acquisition.

In addition to preferential spacer acquisition from pCas, we also observed biased spacer acquisition within individual plasmids and genome sources by mapping the distribution of protospacers. The protospacers present within the *E. coli* BL21 (DE3) genome exhibited a slightly bell-shaped distribution centred at the chromosomal origin of replication (*oriC*), with the presence of hotspots detected in the vicinity of the chromosomal replication terminus (*Ter*) (Supplementary Fig. S7A). Similar distributions have been previously reported in an *E. coli* type I-E CRISPR system [66]. Interestingly, we also observed an additional bell-shaped peak around 750 kb of the genome (Supplementary Fig. S7A), corresponding to the location of the lysogenized DE3 prophage, which allows controlled expression of T7 RNA polymerase (Supplementary Fig. S7A). We repeated the spacer acquisition assay in *E. coli* BL21 (AI), which was engineered by inserting a T7 RNA polymerase into the *araB* locus of the *araBAD* operon instead of using DE3 phage. While the unique protospacer distributions around the *oriC* and *Ter* sites are similar, the additional prophage peak was no longer detected (Supplementary Fig. S7B). Therefore, we suggest that the prophage in BL21(DE3) is induced by the stress condition upon overproduction of *cas* genes and becomes a major source of spacer acquisition in the *E. coli* genome. In addition, we observed several stochastic hotspots of protospacer sites predominantly located in CDS regions (Supplementary Table S4), suggesting that these genes may also be highly expressed under our experimental conditions. For the spacers mapped on pCas and pCRISPR, we also observed a skewed distribution with a considerably higher acquisition rate near the origin of plasmid replication initiation (*ori*) sites (Supplementary Fig. S7C). These distribution patterns were very similar in bacteria carrying different combinations of *cas* genes, suggesting that *ori* is a hotspot for acquisition regardless of the presence of Cas12. This preference for termini of plasmid replication acquisition has been previously reported in the type I-E [66], type I-F [43, 45], type II-A [68], and type III systems [69].

Furthermore, we examined the length distribution of the acquired spacers and found that the spacer length mainly ranged from 27 to 29 bp with the most frequent spacer size being 29 bp; no significant difference was detected upon addition of Cas4 or Cas12 (Fig. 5 and Supplementary Fig. S6D). These results are highly consistent with what we observed from the *in vitro* integration assay (Fig. 3B), proving that spacer size selection is determined only by Cas1 and Cas2.

### Cas4 is required for PAM selection and functions as a 3′ to 5′ exonuclease

From the *in vivo* spacer acquisition assay of the *F. novicida* type V-A system, we did not observe major differences in spacer acquisition efficiencies or in the pre-spacer source when *cas4* is absent or carries a mutation (D78N or L6^STOP^) (Fig. 5 and Supplementary Fig. S5B), which is consistent with previous cas*4* deletion studies of other CRISPR–Cas types [27, 29]. However, the function of Cas4 in spacer acquisition becomes apparent when analysing the PAM sequences of the newly acquired spacers. The PAM sequence located upstream of the protospacer has a conserved motif of 5′-TTTN-3′ (Fig. 5F), as reported previously [70–72, 73]. This motif was only detectable in the strain carrying wt *cas4* (Fig. 5F and Supplementary Fig. S6D), indicating that Cas4 is required for PAM-dependent spacer selection in the type V-A CRISPR–Cas system.

**Figure 6.**
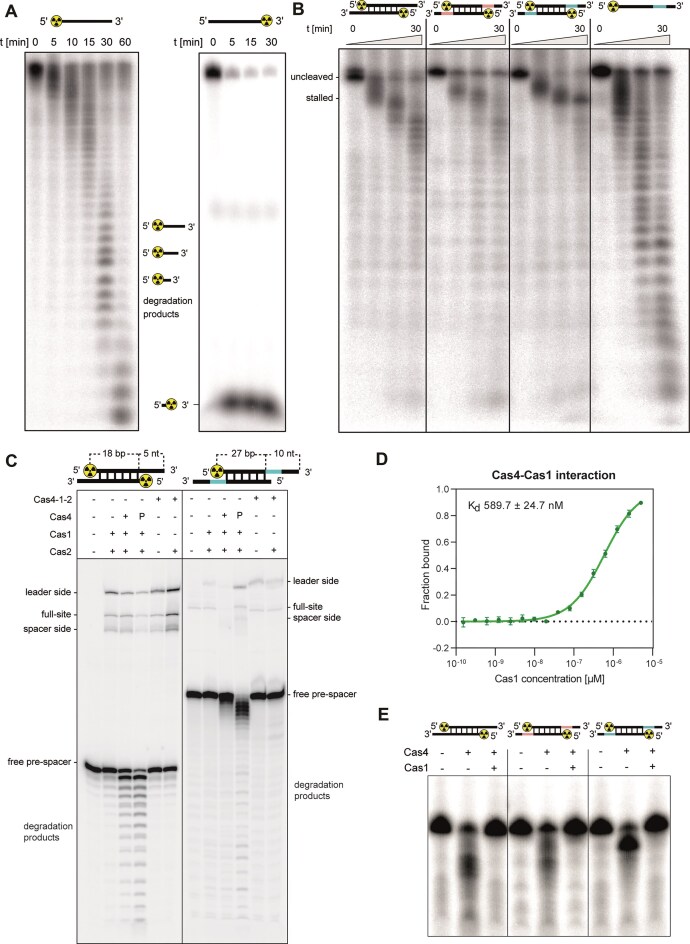
Cas4 is a 3′ to 5′ exonuclease. (**A**) Nuclease activity of Cas4 was analysed using ssDNA substrate labelled with ^32^P at the 5′ or 3′ end. DNA and protein were incubated at 18°C for 0–60 min. Shown are representative gels of three replicates. (**B**) Cas4 and dsDNA or ssDNA substrates containing 10-cytidin-monophosphate single-stranded 3′ overhangs at both ends were incubated at 18°C for 0–30 min. The overhangs were unmodified (black) or contained either the PAM (5′-TTTC-3′; pink) or a PAM-complementary (cPAM, 5′-AAAC-3′; blue) sequence. All DNA substrates were ^32^P-labelled at the 5′ end. Shown is a representative gel of three replicates. (**C**) The *in vitro* integration efficiency of Cas1 and Cas2 was evaluated in the presence of Cas4 using a hairpin CRISPR substrate, a 5′ end ^32^P-labelled pre-processed pre-spacer (left) or an unprocessed pre-spacer containing a PAM-complementary (cPAM, 5′-AAAC-3′, blue, right) sequence. Integration assays were conducted as described in the methods section using either separately purified proteins (final concentrations: 1 µM Cas1, 10 µM Cas2, 5 µM Cas4) or co-purified Cas4–Cas1–Cas2 (final concentration: 75 ng/µl). Proteins were mixed with pre-spacer substrates and incubated at 18°C for 30 min before addition of the CRISPR substrate. Reactions continued at 30°C for 30 min. ‘P’ indicates pre-incubation with Cas4. Here, only Cas4 was incubated with the pre-spacer at 18°C for 30 min before Cas1, Cas2, and the CRISPR substrate were added and incubation was continued at 30°C for 30 min. Shown is a representative gel of three replicates. (**D**) MST analysis of Cas1 and Cas4 interaction. RED-NHS labelled Cas4 was incubated with serial dilutions of unlabelled Cas1. Binding was measured at 23°C and dose response curves were calculated based on MST on time 1.5 s. Error bars indicate the standard deviation of three replicates. (**E**) Cas1 antagonizes the enzymatic activity of Cas4. Cas4 alone (5 µM) or Cas4 and Cas1 (1 µM) were incubated with different dsDNA substrates containing 10-cytidin-monophosphate single-stranded 3′ overhangs at both ends at 18°C for 0–30 min. The overhangs were unmodified (black) or contained either the PAM (5′-TTTC-3′; pink) or cPAM (5′-AAAC-3′; blue) sequence. All DNA substrates were ^32^P-labelled at the 5′ end. Shown is a representative gel of three replicates.

To investigate the enzymatic properties of Cas4, the protein was expressed and purified from *E. coli* and the ability of Cas4 to degrade DNA was evaluated using end-labelled ssDNA. When Cas4 was incubated with 5′-labelled ssDNA, we observed the generation of DNA ladders over time (Fig. 6A), indicating that Cas4 is either a non-specific endonuclease or a 3′ to 5′ ssDNA exonuclease. In comparison, only a single low molecular weight band was detected when ssDNA was labelled at the 3′ end (Fig. 6A), strongly suggesting a 3′ to 5′ exonucleolytic activity of Cas4. We observed that detectable Cas4 nuclease activities was only achieved at relatively high protein concentrations (Supplementary Fig. S8A), consistent with previous reports on Cas4 homologs from *Pyrobaculum calidifontis* [74], *Leptospira interrogans* [75], and *Methanosarcina mazei* [76], which also required micromolar enzyme levels for robust ssDNA cleavage. To verify that the observed activity is intrinsic to Cas4, we constructed two Cas4 mutants, Cas4 ^C16A^, and Cas4 ^D78N^, targeting conserved residues required for Fe-S cluster coordination and nuclease Mg^2+^/Mn^2+^ binding, respectively (Supplementary Fig. S9) [74]. The Cas4 D78N mutation completely abolished ssDNA nuclease activity (Supplementary Fig. S8A), confirming the key role of D78 in metal binding and catalysis. In comparison, attempts to purify Cas4 ^C16A^ failed, as this mutant was found exclusively in the insoluble fraction, indicating that an intact Fe-S cluster is essential for Cas4 structural stability. Consistently, excluding reducing agents from the Cas4 reaction assay abolished nuclease activity (Supplementary Fig. S8A). Together, these findings suggest that both the Fe-S cluster and a reducing environment are critical for maintaining Cas4 activity and may partially explain the relatively low activity of Cas4 *in vitro*.

Next, the ability of Cas4 to degrade dsDNA was assessed. We designed a double-stranded substrate containing single-stranded 3′ ends. We observed degradation of the single-stranded ends, resulting in the accumulation of dsDNA intermediates (Fig. 6B, left panel). Upon prolonged incubation, the intermediates were degraded, indicating that Cas4 either displays a mild cleavage activity on dsDNA or is able to unwind the double-stranded substrate and degrade the resulting single strands. To rule out the possibility that Cas4 harbours any endonucleolytic activity, the protein was incubated with circular ssDNA or dsDNA of phage M13 genome, for which no apparent DNase activity of Cas4 was observed (Supplementary Fig. S8B), strengthening the hypothesis that Cas4 is a single-strand specific exonuclease.

To further analyse whether the enzymatic activity of Cas4 is affected by the presence of the type V-A PAM sequence (5′-TTTN-3′), we used DNA substrates with single-stranded 3′ ends containing either the PAM or the PAM-complementary (cPAM) sequence (Fig. 6B). While there was no difference in the cleavage rate of the whole substrate containing either the genuine PAM or no PAM, we observed a stalled degradation of the dsDNA substrate containing the cPAM motif overhang (Fig. 6B). This suggests that the activity of Cas4 is sequence dependent and that its nuclease or unwinding activity is stalled at the PAM-complementary motif. However, the presence of the cPAM on an ssDNA substrate did not affect the nuclease activity of Cas4, indicating that the motif rather affects its unwinding activity (Fig. 6B, right panel).

Finally, we observed that Cas1 and Cas4 formed a complex using MST with an affinity of 589.7 nM (Fig. 6D), indicating that Cas4 functions together with the Cas1–Cas2 complex to carry out PAM selection during spacer integration. AlphaFold modelling [65] suggests that Cas4 docks on one wing of the Cas1 dimer. In this arrangement, the Cas4 Fe-S cluster is oriented towards Cas1, whereas the catalytic metal-binding pocket of Cas4 is exposed on the opposite side (Supplementary Fig. S9B). In order to analyse whether Cas4 is involved in pre-spacer processing and integration, we repeated the integration assay using a protein mixture of Cas4, Cas1, and Cas2, either separately purified or co-purified and observed that Cas4 did not alter the integration behaviour of Cas1–Cas2 when a pre-processed pre-spacer was used (Fig. 6C). Excess Cas4 or pre-incubation of the pre-spacer with Cas4 led to degradation of the DNA substrate, but the nuclease activity of Cas4 seemed to be antagonized when the co-purified Cas4–Cas1–Cas2 complex was used (Fig. 6C). This observation was further confirmed by incubating Cas1, Cas4, and DNA substrates together. In the presence of Cas1, the nuclease activity of Cas4 was completely abolished (Fig. 6E) and this was also observed for substrates containing the PAM and cPAM sequences (Fig. 6E). These results indicate that either Cas4 interacts directly with Cas1 and displays altered enzymatic properties when embedded in a heteromeric complex, or that Cas1 occupies the DNA substrates and protects them from Cas4-mediated degradation.

We also evaluated the ability of Cas4 to process a pre-spacer substrate in a cPAM-dependent manner by using an unprocessed pre-spacer consisting of a 27 bp DNA fragment and 10 nt 3′ overhangs containing cPAM on both sides (Fig. 6C, right panel). Interestingly, we observed integration bands for the co-purified Cas4–1–2 complex or when Cas4 was pre-incubated with the substrate but not when Cas4 was added at the same time as Cas1 and Cas2 (Fig. 6C, right panel, lanes 3 and 4). This may indicate that Cas4 is responsible for pre-processing the pre-spacers supported by sensing cPAM and subsequent coordination with Cas1–Cas2 to achieve a spacer integration event. Further analysis is required to determine the exact nature of the integration products observed in samples where the pre-spacer was subjected to Cas4-mediated processing prior to integration.

## Discussion

### Spacer integration by Cas1 and Cas2 *in vitro*

A number of studies have provided insights into the mechanisms of spacer integration by Cas1–Cas2, describing sequence requirements of pre-spacers and the CRISPR array for site-specific integration [12]. However, these studies are limited mainly to type I and type II CRISPR–Cas systems.

The most striking difference between these systems is the involvement of non-Cas cellular factors that aid or complement specific reactions during spacer acquisition, which appears to be conserved predominantly in type I systems [12]. The Cas1–Cas2 integrase of these systems recognizes certain parts of the repeat sequence using yet unknown factors in types I-A, I-D, and I-G [30, 57, 58], or proteins such as IHF in types I-E and I-F, which shift the affinity of the integration site towards the leader end of the CRISPR array [44, 56].

In contrast, the type II-A Cas1–Cas2 complex displays an intrinsic affinity for the leader-proximal repeat by recognizing a small motif termed the leader-anchoring site and thus, does not rely on additional factors for site-specific spacer integration [16]. In this study, we obtained comparable results for the type V-A integration proteins Cas1 and Cas2 *in vitro*, since they also facilitate the recognition of the leader-repeat junction without the need for supplementary factors. While no more than three nucleotides of the leader seem to be important for site-specific integration *in vitro*, Cas1 displayed a strong preference for a 4-nucleotide palindromic sequence present at both ends of the repeat. Cas2 was required for the determination of the second integration site at the repeat-spacer boundary and hence, is essential for full-site spacer integration. The terminal sequences and overall length of the repeat are essential for proper recognition and full-site integration of new spacers. Base deletions or additions to the repeat decreased the occurrence of full-site products, suggesting that spacer integration also relies on a ruler mechanism in type V-A CRISPR–Cas systems. Our results indicate that the mid-repeat sequence of the array appeared rather negligible for recognition by Cas1 and Cas2. It is noteworthy that small successive sequence alterations of the repeat might be tolerated more easily by Cas1–Cas2 compared to larger simultaneous mutations, which was previously demonstrated for type II-A systems, thus highlighting the relevance of the mid-repeat region [17, 62].

CRISPR repeats usually harbour palindromic motifs that can vary in length and location within the repeat. Their roles in the spacer integration process differ among CRISPR–Cas types. In types I-E and II-A, the CRISPR repeats were shown to be necessary for recognition by Cas1 and Cas2 [17, 19], whereas in type I-A only the leader-repeat junction is critical for spacer integration [30]. In addition to both repeat ends, the mid-repeat region was shown to be important for proper integration in subtypes I-G and I-B, further demonstrating the diverse sequence requirements of homologous Cas1–Cas2 integrases [58, 77].

The type V-A repeat contains two short palindromic motifs, which are located throughout the repeat. However, only mutations in the terminal palindromes TCTA had an effect on spacer integration, with the leader-proximal site being essential for integration. Surprisingly, base substitutions at repeat positions 13–18 increased the amount of full-site integration events (Fig. 4E). Currently, we do not have an explanation for this observation, but we speculate that our substitutions might have increased the affinity of the integrase for the repeat sequence. The introduction of other nucleotides in that region may not change the integration efficiency. It would be interesting to investigate whether specific base alterations within the repeat would also cause an increased spacer incorporation rate *in vivo*. This could become especially interesting when utilizing the Cas1–Cas2 integrase as a molecular recording device within cells—an application that has been used recently to store information inside bacterial genomes or to record transcriptional events in a timely manner [53, 78, 79]. However, these applications are restricted by the overall low integration efficiencies of Cas1–Cas2 that are frequently observed *in vivo* [12]. Defining properties that increase spacer integration efficiency might help to improve such applications.

In this study, we also aimed to understand the role of Cas2 and its underlying mechanism. We observed that a conserved D7 residue of Cas2 is critical for spacer integration *in vivo*. Biochemical analyses further revealed that the Cas2 D7N mutation does not affect the binding of Cas2 to Cas1, but causes a defect in Cas2 dimerization. Meanwhile, the Cas1–Cas2 (D7N) complex still displayed WT levels of leader half-site integration products, whereas the occurrence of full-site products was significantly reduced *in vitro*, indicating impaired guiding of the pre-spacer substrate to the second integration site. This observation suggests a different integrase property compared to the type I-E Cas1–Cas2 complex of *E. coli*, where the equivalent residue of Cas2 (a glutamate at position 9) had no effect on the integration efficiency of the integrase *in vivo* or *in vitro* [13, 14]. The AlphaFold structure model provides further evidence that the D7N change alters the dimer structure of Cas2, burying the negatively charged channel in between. This structural change potentially blocks metal binding and could interfere with the flexibility of Cas2 as a guide for Cas1 to perform full-site integration, thereby disrupting the ruler mechanism of the Cas1–Cas2 complex.

Furthermore, we provide evidence that the D7 residue of Cas2 is involved in the coordination of Ca^2+^, Cas2 dimerization and the enhancement of full-site spacer integration by the Cas1–Cas2 complex *in vitro*. It is worth noting that the WT Cas2 protein also shows a lower affinity for Mg^2+^ than for Ca^2+^ (Supplementary Fig. S3A), promoting a higher rate of full-site integration in the absence of Ca^2+^ compared to the D7N variant (Fig. 2A). Given that the Ca^2+^ concentrations used in this study (5–10 mM) far exceed free intracellular Ca^2+^ (low µM level), while free Mg^2+^ is present at low mM levels, Mg^2+^ is expected to occupy primarily the D7 site in cells. Structural studies of the type II-A Cas1–Cas2 complex in *Enterococcus faecalis* have reported that the equivalent aspartate of Cas2 coordinates a magnesium ion that makes direct contact with the pre-spacer [62]. Accordingly, we propose that the type V-A Cas2 dimer forms a negatively charged channel for metal ion coordination at D7, allowing a stretchable Cas1–Cas2 complex to guide Cas1 in positioning the pre-spacer at the leader-distal integration site, with Mg^2+^ serving as the physiological cofactor *in vivo* and Ca^2+^ providing additional stimulation under high Ca^2+^ conditions *in vitro*. It is noteworthy that intracellular Ca^2+^ levels in bacteria can increase substantially under certain stress conditions and specific triggers [80, 81], suggesting that calcium might play a regulatory function in the type V-A CRISPR adaptation process in *F. novicida in vivo*.

### Role of Cas12 in spacer acquisition *in vivo*

Although type V-A Cas1 and Cas2 are sufficient for spacer integration *in vitro* when using a properly processed substrate, we observed only low levels of spacer acquisition *in vivo* in the absence of Cas12 using *E. coli* as a heterologous host. Our large-scale *in vivo* spacer acquisition screen revealed that the mature interference complex plays a role in increasing the efficiency of naïve adaptation in the *F. novicida* type V-A CRISPR–Cas system. We observed that the Cas12 DNA cleavage residue E1006 in the RuvC domain [71, 70], the PAM-interacting residue K671 [72], and the presence of crRNA are all required for efficient spacer acquisition. A comparable observation was made in the type I-F system of *Pseudomonas aeruginosa*, where a fused Cas1–2/3 complex can produce only low levels of spacer acquisition, but the presence of the Csy effector complex and crRNA enhances adaptation efficiency [43–45]. In the type I-B system of *Haloarcula hispanica* and *Pyrococcus furiosus*, loss of either effector proteins or Cas3 inhibited adaptation [37, 38]. In the type II-A system of *Streptococcus thermophilus* and *Streptococcus pyogenes*, tracrRNA–Cas9 has been shown to be essential for PAM-dependent protospacer selection of invading DNA [35, 36]. However, the nuclease domain of Cas9 was found to be dispensable [36], suggesting that Cas9 may play a different role in spacer acquisition.

Although Cas12 could significantly accelerate the removal of spacer-targeted DNA substrates, leading to the accumulation of spacers that favour bacterial growth, the high rate of spacer acquisition was also observed in the absence of Cas4. This suggests that Cas12 may have a broader function beyond PAM-dependent interference. Cas12 has been shown to mediate PAM-dependent *cis*-cleavage of dsDNA and PAM-independent *cis*- and *trans*-cleavage of ssDNA [82–84]. Recently, Cas12 nicking of dsDNA was reported for mismatched *cis*-target or non-target in *trans* upon activation, depending on substrate type and topology [85–88]. With respect to the CRISPR adaptation process, it is plausible that these *trans*-dsDNA and ssDNA nicking and cleavage activities are utilized for endonucleolytic cuts on potential substrates. On the one hand, this could create multiple entry points in the protospacer regions to allow access of Cas4 and/or the adaptation complex, and on the other hand, the cleavage activity may pre-process the pre-spacers to the appropriate sizes. The unwinding function of Cas12 [72] might also be involved in efficient spacer acquisition, as its conserved residue K671 in the PAM interacting domain was observed to be essential. A similar mechanism was suggested in a study of type I-B spacer acquisition, where the effector complex interacts with adaptation modules and cleaves endogenous, crRNA-independent R-loops *in vivo*, potentially helping the Cas1–2–4 complex to convert the nicked DNA into new spacers [37].

Surprisingly, a recent study on type V-A and V-B adaptation claimed that Cas12 is not essential for spacer acquisition [59], which is not consistent with our observations. This discrepancy may be attributed to the different experimental setups employed in the two studies. Specifically, the antibiotic selection pressure for plasmids was maintained after *cas* gene induction, and instead of a direct amplification process, the authors used a population PCR approach with forward primers containing a single 3′ nucleotide mismatch [59]. This approach allows preferential amplification of expanded arrays and enhances both band intensity and the number of spacers detected. However, new spacers initiated with the same nucleotide as the pre-existing spacer elude detection, and quantitative comparisons between different genotypes are also compromized.

It is important to note that in our *in vivo* experimental setup, efficient spacer acquisition requires not only a functional adaptation module and crRNP, but also the presence of suitable Substrates. In other words, the vast majority of newly acquired spacers are originating from the pCas plasmids encoding *cas* genes (Fig. 5E and Supplementary Fig. S6B and C). The strong preference for spacer acquisition from heterologous plasmids has been previously detected [66, 43, 45] and explained by a higher density of Chi sites on the host chromosome and a greater number of replication forks on the foreign DNA [66]. Although this provides a partial explanation for our observations of spacers originating from pCas plasmids and the DE3 prophage region of the *E. coli* genome, it becomes challenging to comprehend with the introduction of an additional high copy number plasmid, designated pFliC. Remarkably, all plasmids employed in our study do not contain Chi sites. Compared to pFliC (F1 and ColE1 origin, ~300–500 copies per cell), both pCRISPR (p15a origin, ~10 copies per cell), and pCas (CloDF13 origin, ~20–40 copies per cell) are relatively low copy number plasmids. However, in the presence of additional pFliC, whether induced or not, a significantly higher rate of spacer acquisition from pCas was still observed. One potential explanation for this phenomenon is that cells replicating without the metabolic burden associated with the adaptation process may gain a growth advantage, leading to the accumulation of spacers targeting plasmids carrying *cas* genes.

A comparable observation was reported in the aforementioned study of type V spacer acquisition *in vivo* [59]. The vast majority of spacers were acquired from adaptation module carrying plasmids, even when a pTarget plasmid containing a protospacer and a canonical PAM was used in addition to pEffector carrying *cas12* and pAdaptation carrying adaptation modules (*cas4-1-2* and CRISPR array) [59]. This finding lends further support to our hypothesis that the origin of spacers is not solely due to enhanced accessibility of MGEs or selective pressure imposed by the expression of toxic proteins (i.e. Cas12), but rather suggests that bacteria may tend to exclude the adaptation modules through an adaptation process. This may also explain previous observations in *Francisella*, where many of the strains lose prior type V CRISPR–Cas systems [89].

### The mechanism of Cas4 dependent spacer acquisition

The *cas4* gene of the type V-A CRISPR–Cas system in *F. novicida* U112 contains a nonsense mutation, which alters the leucine L6 TTG codon to a TAG stop codon. This observation led us to hypothesize that Cas4 in strain U112 is translated as a shorter version of 21 amino acids from an alternative start codon downstream of TTG. The Cas4 protein contains an Fe-S cluster conjugated to 4 conserved cysteines, which often plays a structural role and presumably supports exo- and endo-nuclease activities [33, 90, 74, 91]. Structural analysis of FnoCas4 shows that the N-terminal conserved cysteine C16 is involved in the Fe-S structure (Supplementary Fig. S9), suggesting that the short version, if it exists, may be functionally affected. This mutation in Cas4 could be generated by genomic pathoadaptation in response to changing bacterial environments, consistent with the loss of pre-existing type V CRISPR–Cas systems observed among *Francisella* strains [89]. Further examination of the native protospacers revealed that spacers 4 and 5 were derived from a temperate bacteriophage possessing the correct PAM sequence TTTN, indicating that Cas4 was functional at the time these spacers were acquired. An alternative explanation for the Cas4 mutation is that the relevant TAG could be suppressed as an amber stop codon for translational read-through to allow the incorporation of non-canonical amino acids. Background amber suppression of TAG has been reported in various strains of *E. coli* [92], signifying that instead of leading to translation termination, an amino acid is incorporated and yields a full-length protein. Further evidence is needed to confirm whether amber suppression occurs in *F. novicida* to compensate for the mutation and produce a functional Cas4 protein.

One of the aims of our study was to understand the mechanism of Cas4 assisted spacer acquisition in type V-A CRISPR immunity. To date, the mechanisms of Cas4 have been reported predominantly in type I CRISPR adaptation, acting either as a 5′ to 3′ [74, 91] or bidirectional exonuclease [90] to unwind dsDNA and trim protospacers, or as a PAM-specific endonuclease [28, 33, 93] to facilitate directional integration of protospacers. However, our *in vitro* experiments indicate a slightly different mechanism. We observed that type V-A FnoCas4 harbours an ATP-independent unwinding activity towards dsDNA and a 3′ to 5′ exonucleolytic activity towards ssDNA substrates. This unwinding activity appears to be stalled at short poly-A regions within partially ssDNA substrates. In accordance with our *in vivo* spacer acquisition assay, FnoCas4 is required for PAM-dependent pre-spacer selection, and strains harbouring FnoCas4 showed enrichment of 5′-TTTN-3′ directly upstream of the protospacer non-targeting strand. This PAM sequence is complementary to poly-A, suggesting that the unwinding activity of FnoCas4 is possibly relevant to its PAM-dependent pre-spacer processing. PAM-dependent 3′ end trimming was also observed in a previous study of type I-A CRISPR–Cas system, in which the PAMs led to extensive DNA processing by Cas4, while poly-T sequences were largely untrimmed [30].

Although the specific 3′ to 5′ exonucleolytic activity of FnoCas4 has never been reported for Cas4 proteins, the host 3′ to 5′ exonucleases DnaQ and ExoT [47, 94] have been shown to play a Cas4-like role, processing pre-spacers to a size suitable for integration. Recent studies of the Cas2-DnaQ fusion protein have provided further elucidation of this mechanism, which trims PAM-containing and PAM-deficient 3′ overhangs with different efficiencies [49, 50]. It has been proposed that in these cases, it is Cas1 that recognizes the PAM or PAM-complementary sequence and protects it from DnaQ trimming [47, 49]. This is in contrast to our observations suggesting that Cas4 is responsible for the stalled unwinding of pre-spacers upon recognition of the cPAM. Furthermore, we have shown that FnoCas4 forms a complex with Cas1 *in vitro* and that this interaction inhibits the DNA processing capacity of Cas4. The tight association of Cas4 and Cas1 has also been observed previously [28, 32, 93, 95] and was further demonstrated by investigating the Cas4–Cas1 fusion protein, which selects PAM-containing pre-spacers and couples the timing of PAM processing to establish integration directionality [33, 34].

**Figure 7.**
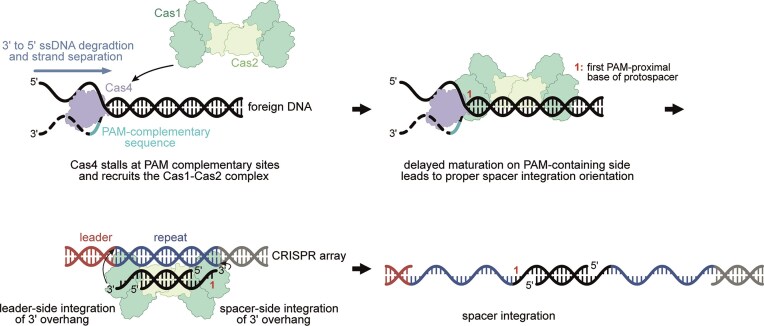
Model of Cas4-mediated spacer integration in the type V-A CRISPR–Cas system.

Therefore, in combination with our observations that excess or pre-incubation of Cas4 with pre-spacers resulted in more integration products compared to the simultaneous addition of Cas4–1–2 (Fig. 6C), we propose a unique exonucleolytic mechanism of Cas4 in the type V-A CRISPR–Cas system: Cas4 unwinds dsDNA and trims the 3′ end of ssDNA substrates until it encounters a PAM complementary sequence that slows down this process. The nuclease recruits the Cas1–Cas2 integrase, which captures DNA fragments of a specific length from further degradation by Cas4. Stalling of Cas4 at cPAM results in a delay in the maturation of the PAM-containing side of the fragments. Thus, the PAM-deficient side of the DNA substrate is trimmed first and integrated into the CRISPR array by Cas1 at the leader-repeat junction, while the PAM-containing side is further processed and forced to integrate at the spacer-side by the Cas1–Cas2 complex to ensure proper directional integration of the pre-spacer into the CRISPR array (Fig. 7).

## Supplementary Material

gkag276_Supplemental_File

## Data Availability

NGS sequencing data are available at the European Nucleotide Archive (accession: PRJEB87316).
