# Supplementary material for: Insights into spacer acquisition of the type V-A CRISPR–Cas system of Francisella novicida U112
Source: Nucleic Acids Res. 2026 Mar 30;54(6):gkag276. doi: 10.1093/nar/gkag276 (PMC13034035; doi:10.1093/nar/gkag276)
Supplement: gkag276_Supplemental_File [file gkag276_supplemental_file.docx]

# SUPPLEMENTARY FIGURES


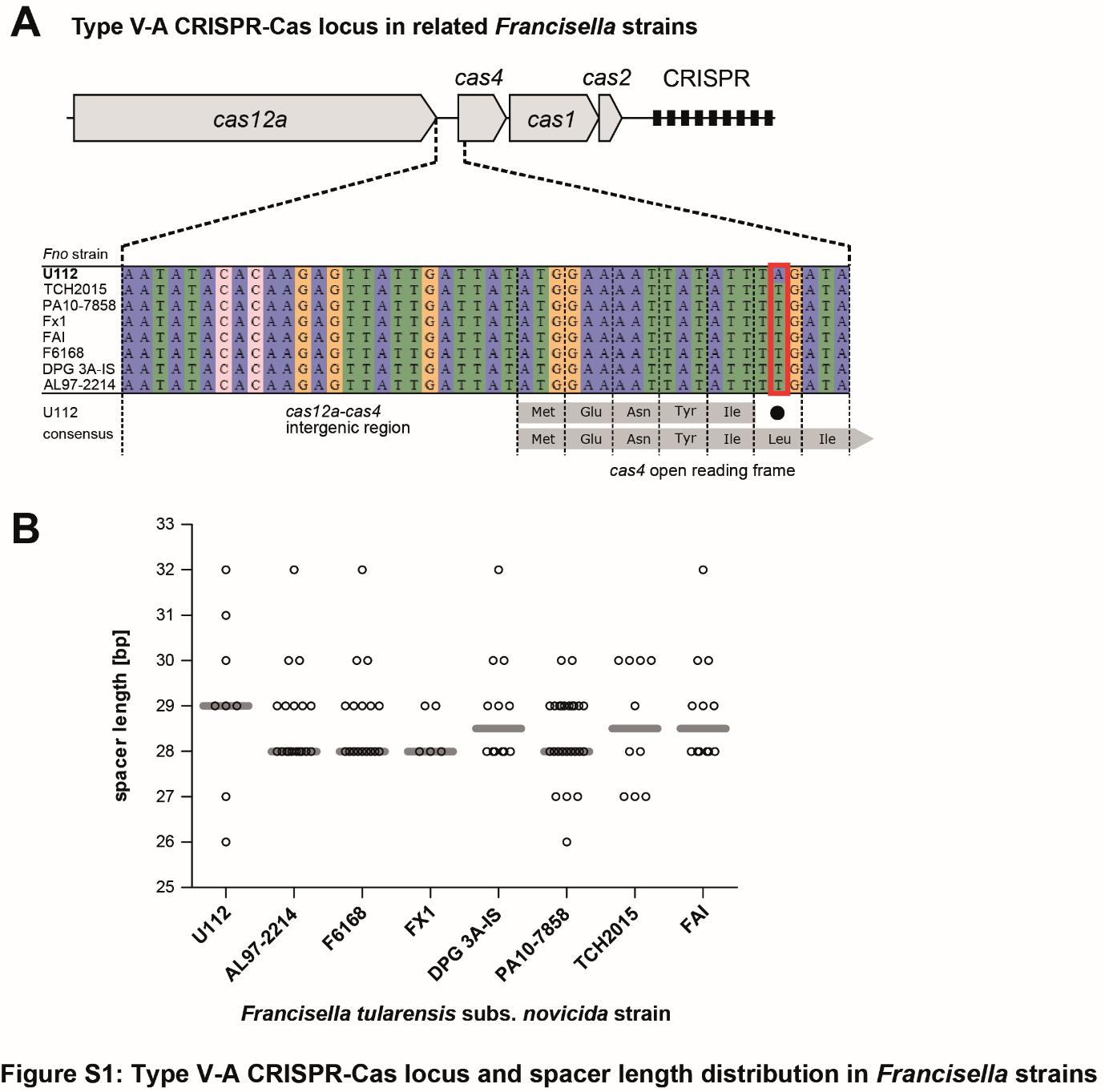


### Figure S1. Type V-A CRISPR-Cas locus and spacer length distribution in *Francisella* strains.

**(A)** Representation of the type V-A CRISPR-Cas system in different *F. novicida* strains. The alignment shows the intergenic region between *cas12a* and *cas4* and part of the open reading frame of *cas4*. The red box indicates a nonsense mutation in the *cas4* gene present in strain U112. The mutation presumably leads to a premature stop during translation of the protein (black sphere). **(B)** Spacer lengths of type V-A CRISPR arrays in different *F. novicida* strains. The grey line indicates the median spacer length.


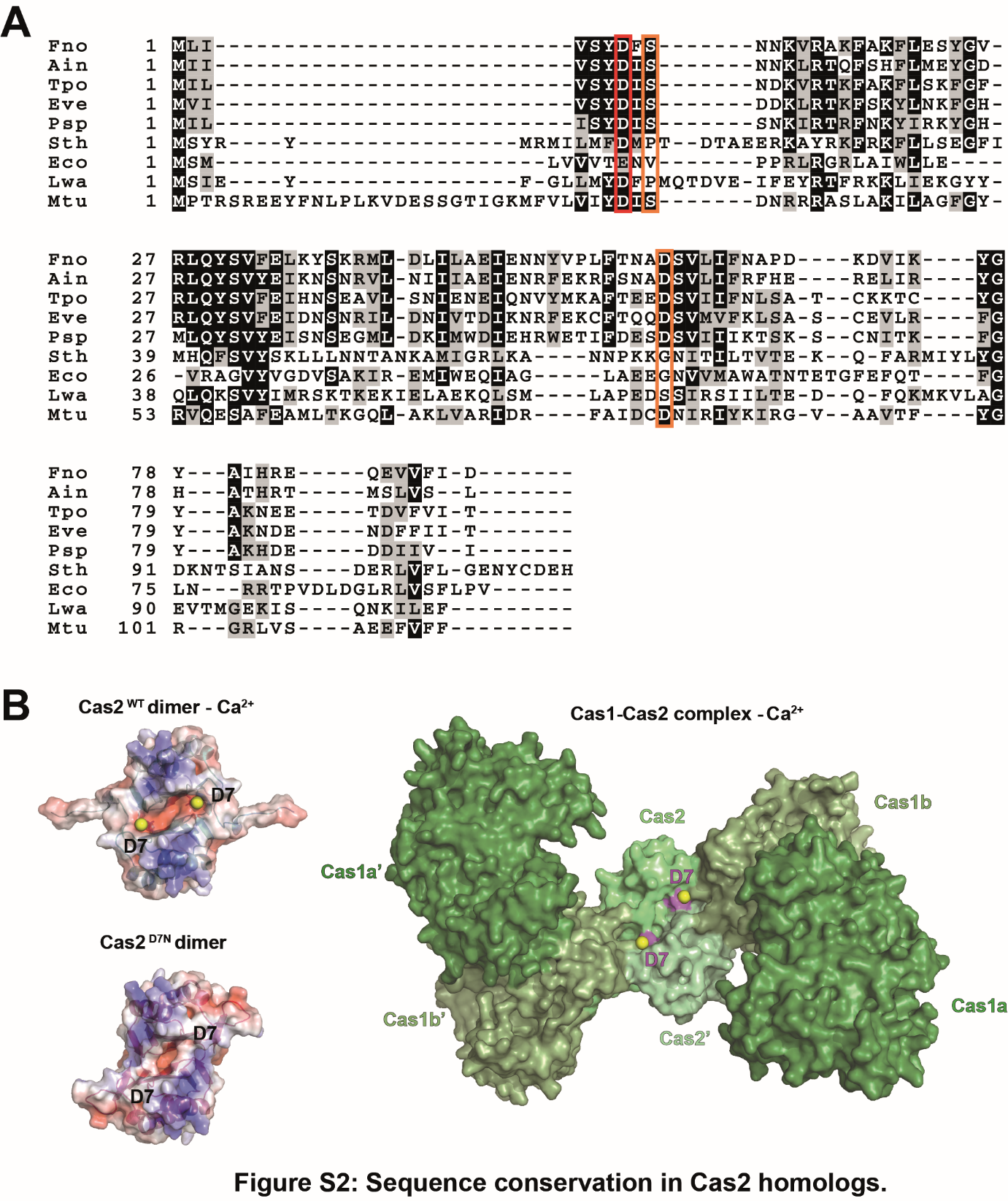


### Figure S2. Conserved aspartate residue of Cas2 facilitates full-site integration.

**(A)** Sequence alignment of multiple Cas2 homologs. Residues mutated in this study are indicated by boxes (red mutation abolishes spacer integration, orange mutation does not abolish spacer integration). Cas2 sequences were derived from: Fno - *Francisella novicida*; Ain - *Acinetobacter indicus*; Tpo - *Treponema porcinum*; Eve - *Eubacterium ventriosum*; Psp - *Proteocatella sphenisci*; Eco - *Escherichia coli*; Lwa - *Leptotrichia wadei*; Mtu - *Mycobacterium tuberculosis*. **(B)** Electrostatic potential map of Cas2 WT or D7N mutant dimers and structure of (Cas1)_4_–(Cas2)_2_ complex with calcium ions. The Ca^2+^ is labelled in yellow and the D7 site on the right panel is highlighted in magenta. Structure complexes were predicted using the AlphaFold server (1).


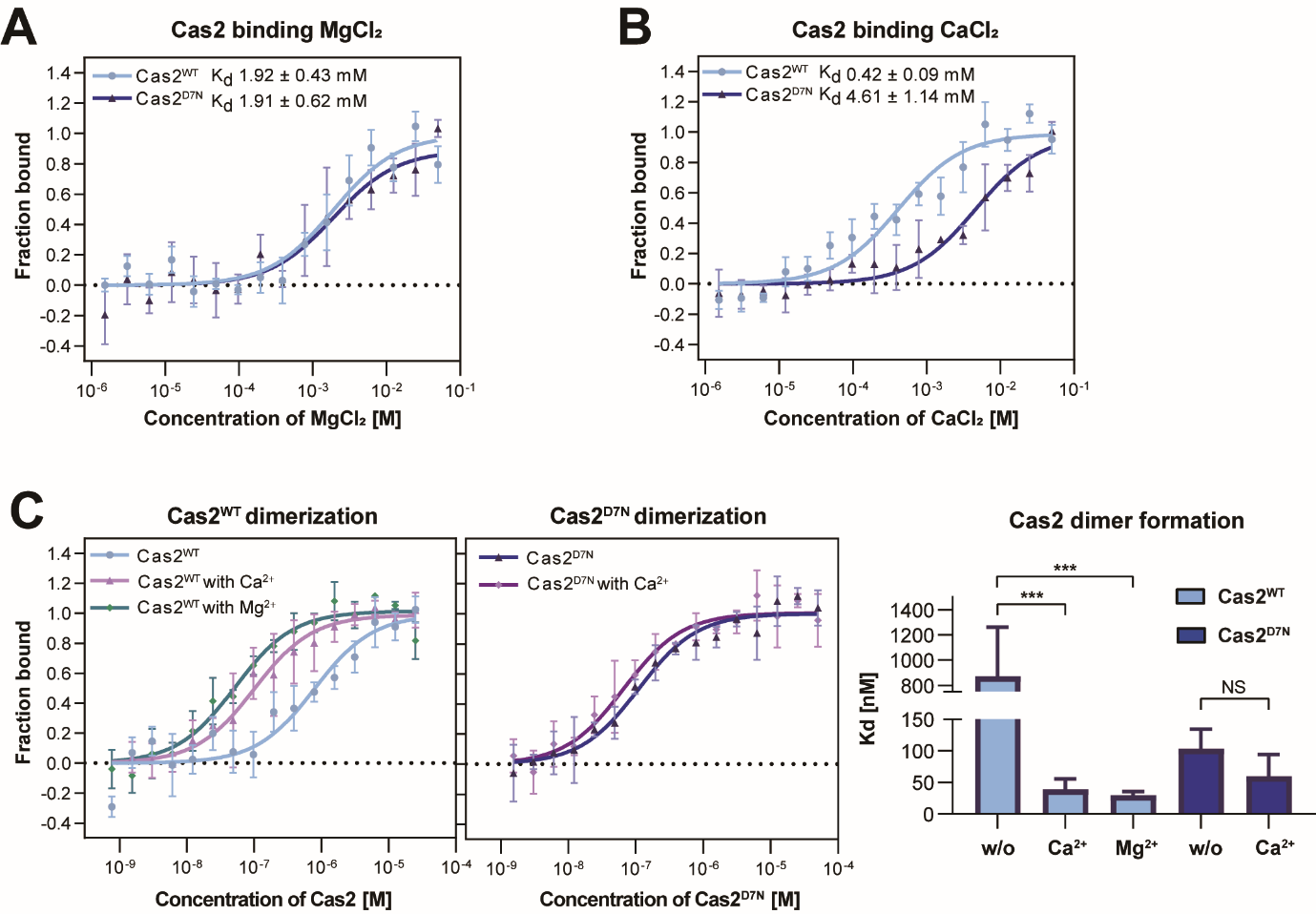


### Figure S3. Divalent metal ion coordination by Cas2.

Microscale thermophoresis (MST) analysis of the interaction between Cas2 and **(A)** MgCl_2_ or **(B)** CaCl_2_. RED-NHS labelled Cas2 WT or Cas2 D7N were incubated with serial dilutions of MgCl_2_ or CaCl_2_, binding was measured at 30°C and dose response curves were calculated based on MST on time 1.5 s or 5 s. Error bars indicate the standard deviation of three replicates. **(C)** MST analysis of Cas2 dimerization without or with MgCl_2_ or CaCl_2_. RED-NHS labelled Cas2 WT or Cas2 D7N was incubated with serial dilutions of unlabelled Cas2 or Cas2 D7N. Binding was measured at 23°C and dose response curves were calculated based on the ligand-induced fluorescence change. Error bars indicate the standard deviation of three replicates. The determined Kd values are plotted on the right. Statistical significance was analysed using one-way ANOVA with Dunnett's multiple comparison test. Asterisks indicate significant changes in dimerization binding affinity, P values were reported in accordance with APA style: P<0.033 (*), P<0.002 (**), P<0.001 (***), NS, not significant.


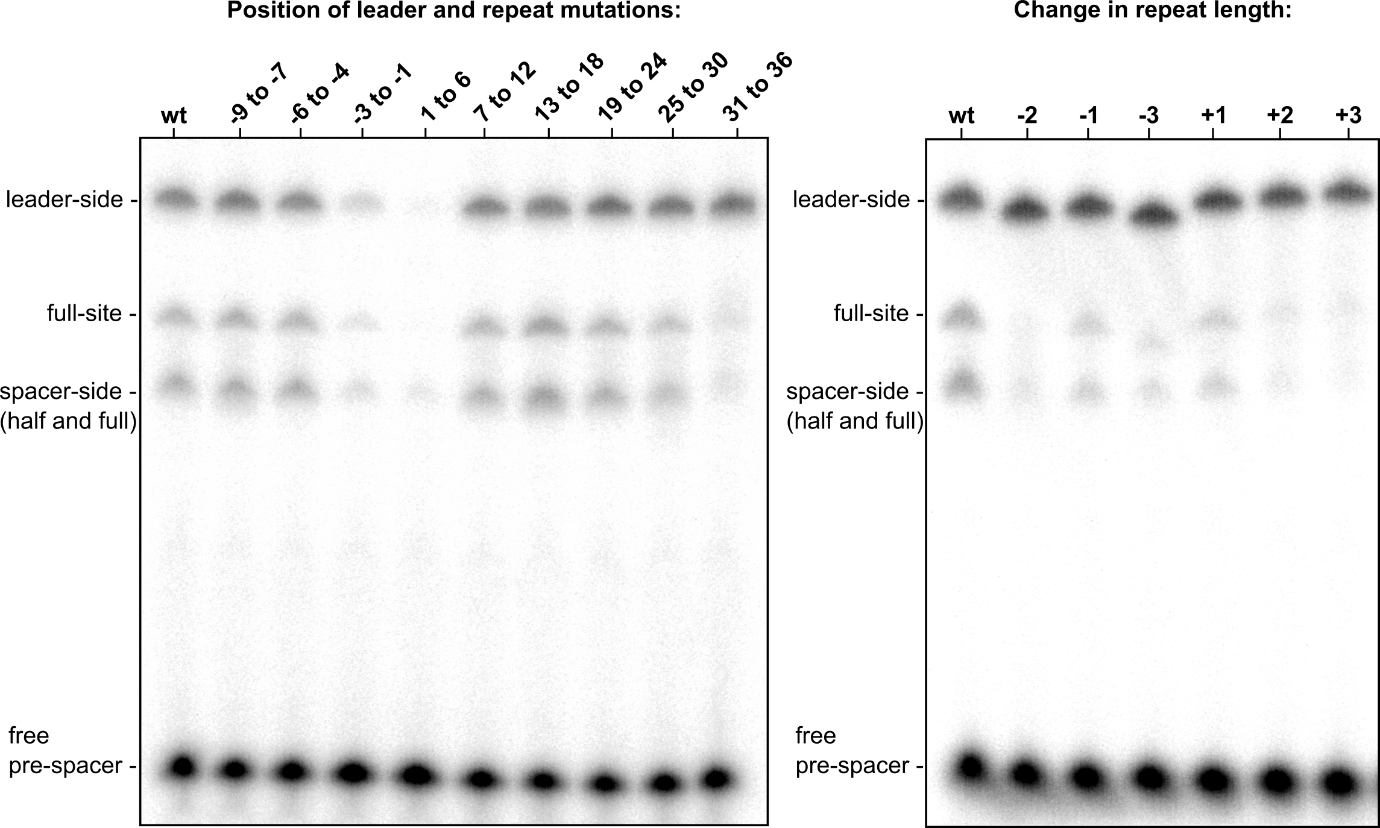


### **Figure S4. Leader-side and full-site integration products on mutated CRISPR arrays.**

The leader and the repeat were mutated in intervals of 3 bp and 6 bp, respectively (indicated by double headed arrows in Figure 4D). See also Table S3 for sequences alteration details. Representative images are shown for three replicates.

###
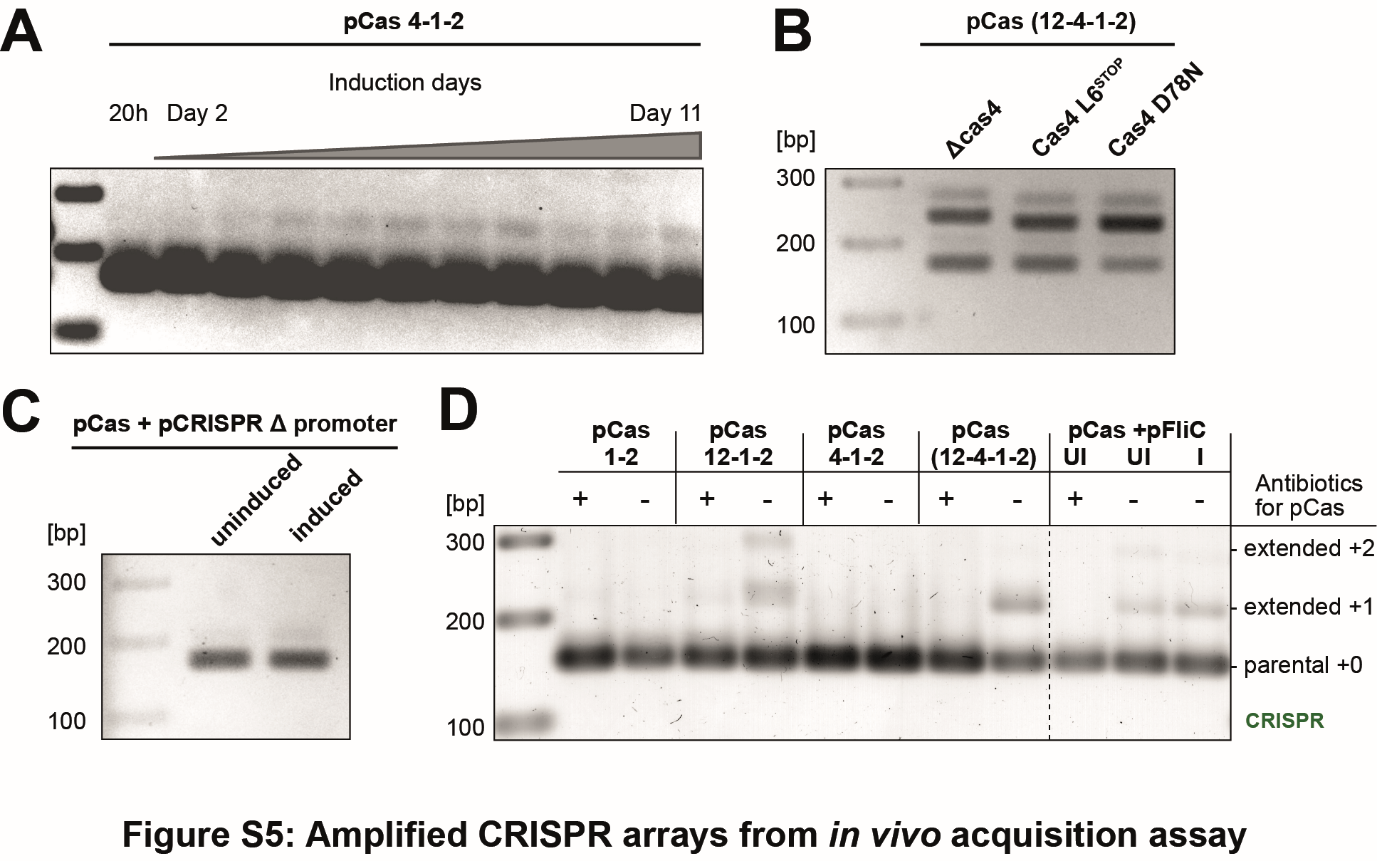
Figure S5. Amplified CRISPR arrays from *in vivo* spacer acquisition assay.

Agarose gels showing amplified CRISPR arrays following *in vivo* spacer acquisition assays, with gene expression induced over an extended period of 11 days using the wt *cas* genes **(A)** or for 20 h using Cas4 mutants **(B)** or variants lacking CRISPR array transcription (Δ promoter) **(C)**. Gene expression was induced with only antibiotic selection for pCRISPR or with antibiotic selection for both pCRISPR and pCas **(D)**. Representative images are shown for at least three replicates.


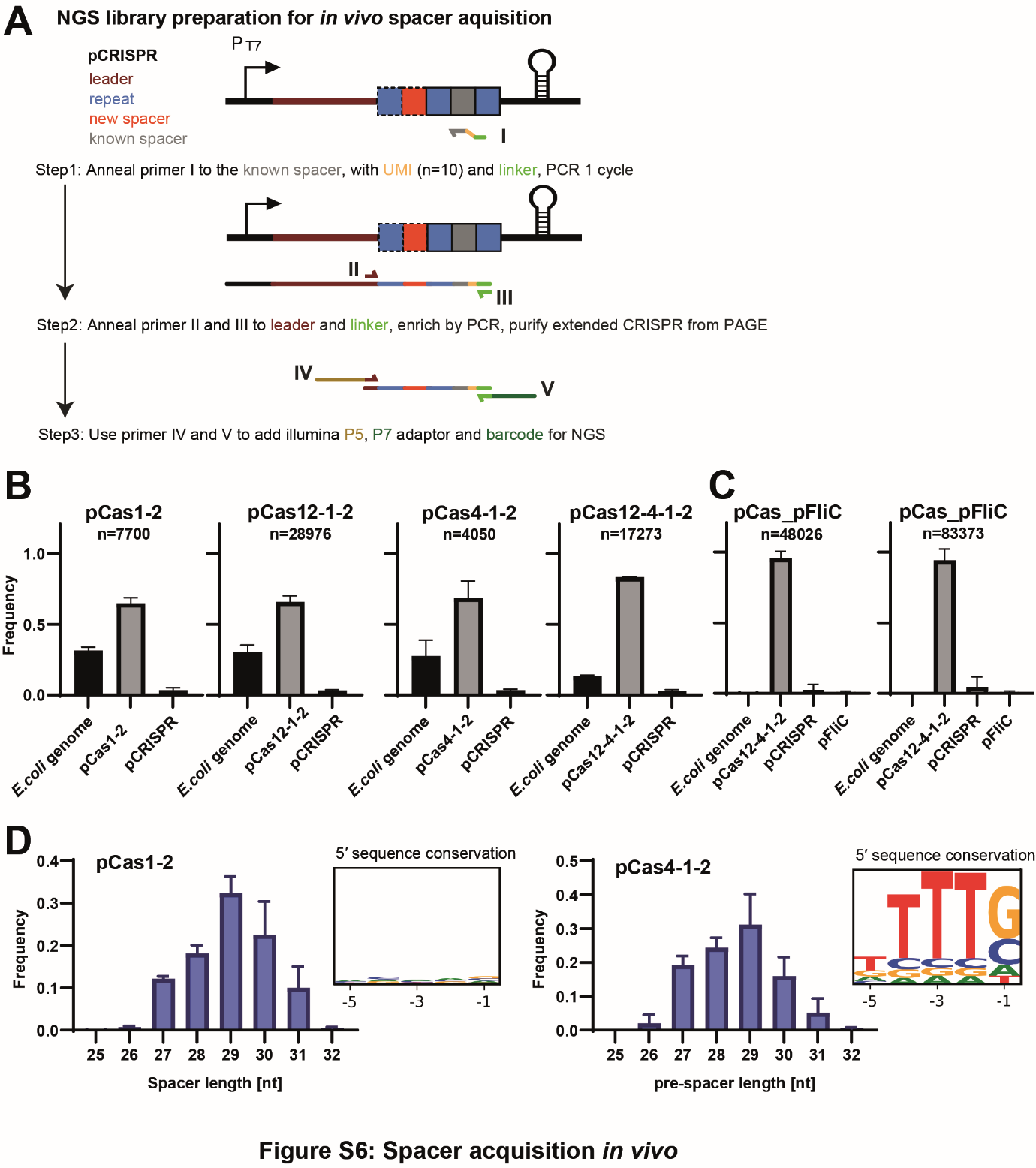


### Figure S6. High-throughput sequencing of acquired spacers *in vivo*.

**(A)** Flowchart of NGS library preparation. **(B)** Relative frequencies of spacers acquired from pCas, pCRISPR or *E. coli* genome following NGS analysis of the amplified CRISPR array after *in vivo* acquisition assay with antibiotic selection for pCas and pCRISPR upon gene expression. Error bars indicate the standard deviation of three replicates. The total numbers of acquired spacers for each strain are indicated. **(C)** Relative frequencies of spacers acquired from pCas, pCRISPR, pFliC or *E. coli* genome following NGS analysis of the amplified CRISPR array after *in vivo* acquisition assay with antibiotic selection for pCRISPR upon gene expression for pCas/pCRISPR alone (left) or pCas/pCRISPR/pFliC (right). Error bars indicate the standard deviation of three replicates. The total numbers of acquired spacers for each tested strain are indicated. **(D)** Length distribution of acquired spacers based on NGS sequencing data. Error bars indicate the standard deviation of three replicates. Logo plots of conserved sequences directly upstream of protospacers acquired *in vivo* in both tested strains are indicated in black boxes (positions -5 to -1 referring to protospacer location).


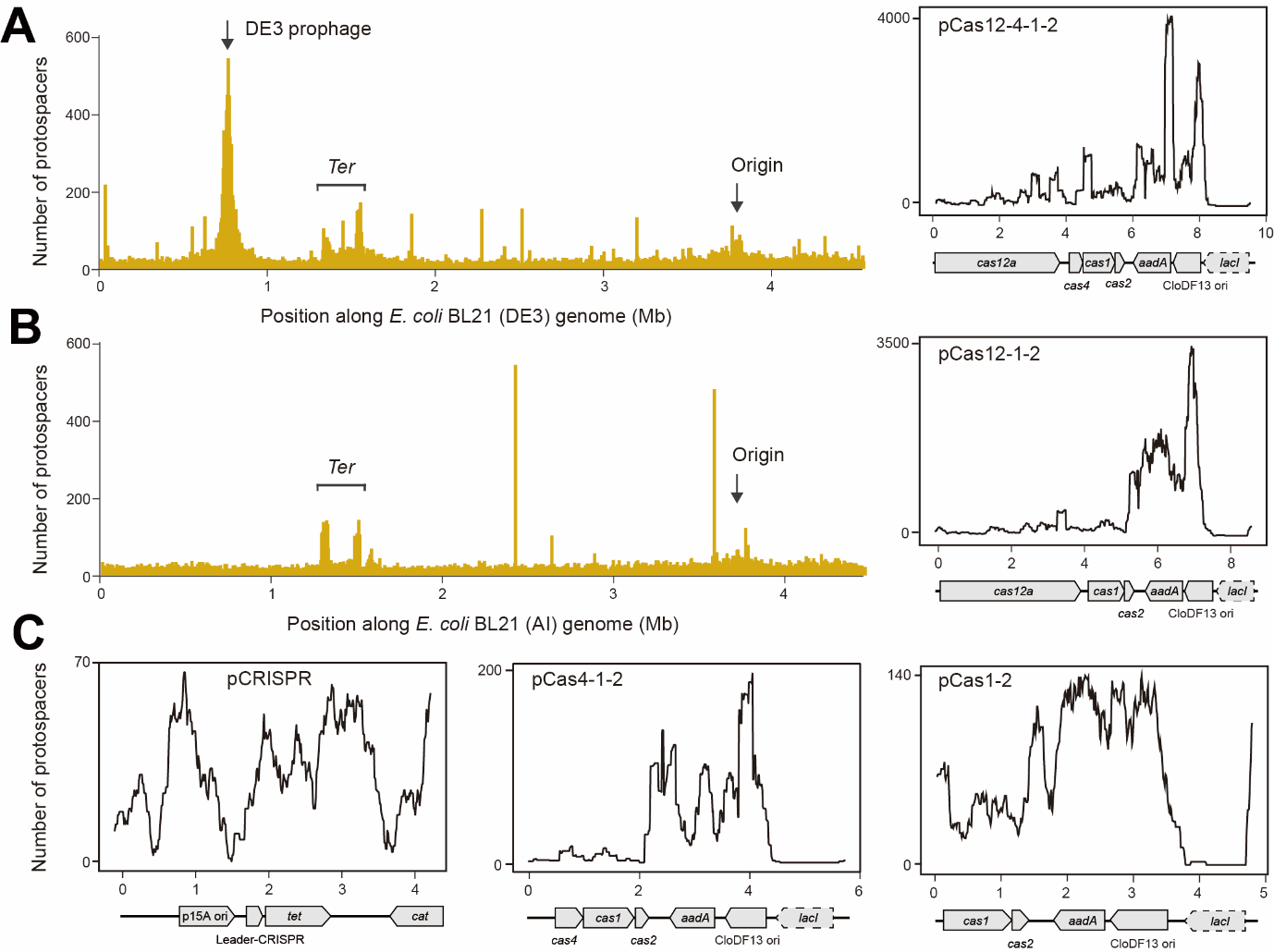


### Figure S7. Mapping of protospacer distribution on *E. coli* BL21 genome and plasmids.

**(A, B)** Distribution of protospacers along the *E. coli* BL21 (DE3, CP001509.3) and BL21 (AI, CP047231.1) genomes. Protospacers were deduced from aligning acquired spacers from pCRISPR after 20 h growth with induction of pCas and pCRISPR and plotted along the genome using a bin size of 5000 bp. The lambda DE3 prophage, chromosomal origin of replication and chromosomal replication terminus (*Ter*) regions are labelled, stochastic hotspots are listed in Table S4. **(C)** The distribution of protospacers along the pCas and pCRISPR plasmids. Data were plotted with a sliding window of 100 bp width and 10 bp step-size and represent the sum of three replicates. For all plots, only protospacers unique to the source are shown, e.g. *lacI* regions shared between plasmids and the *E. coli* genome were excluded from the mapping analysis; regions shared within a single source were averaged by spot number.


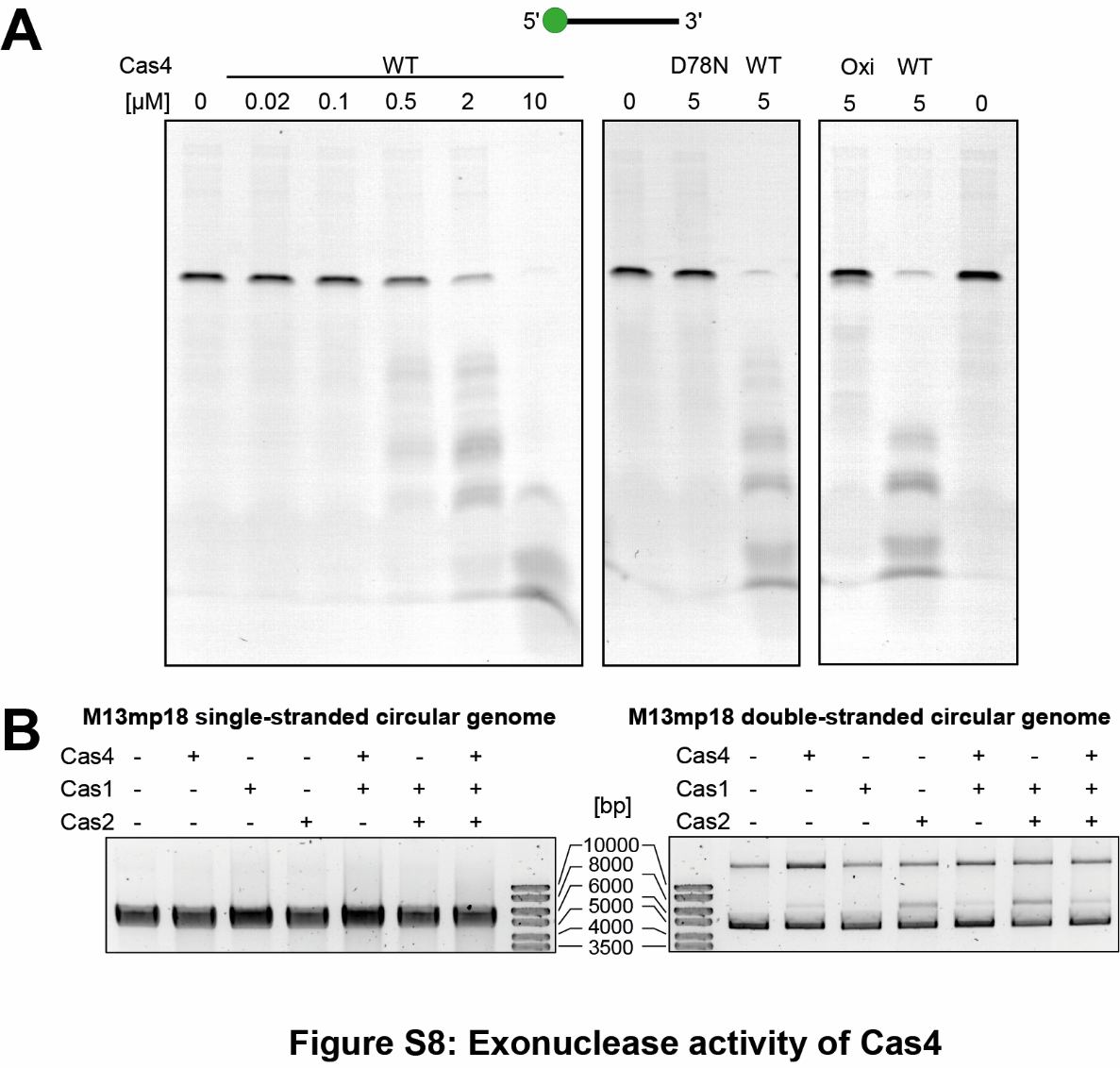


### Figure S8. Exonuclease activity of Cas4.

**(A)** Nuclease activity of Cas4 and Cas4 ^D78N^ was analysed using ssDNA substrate labelled with 6-FAM at the 5′ end. 200 nM DNA and 0-10 µM protein were incubated at 18°C for 30 min. 'Oxi' indicates conditions in which WT Cas4 was desalted into a DTT-free buffer and subsequently assayed in a reaction buffer lacking DTT. Shown are representative gels of three replicates. **(B)** The endonuclease activity of Cas4 was tested using circular single-stranded (upper panel) and circular double-stranded (lower panel) M13mp18 phage genome. Cas1 and/or Cas2 were included in the reactions as indicated. Reactions were analysed on a 0.8% agarose-TAE gel and visualized by ethidium bromide staining. Shown are representative gels of three replicates.


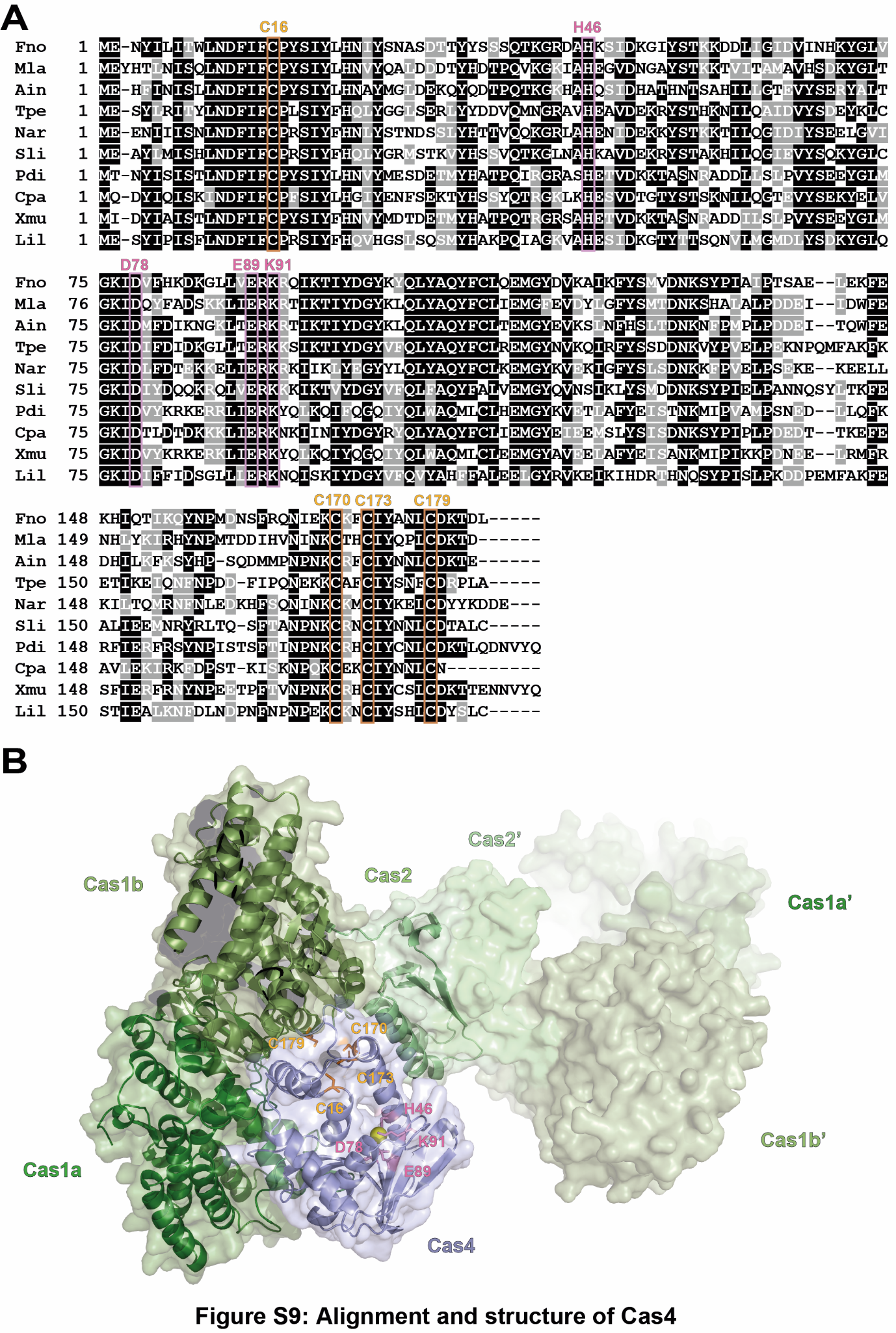


### Figure S9. Sequence alignment of Cas4 and structure model of Cas4-1-2.

**(A)** Sequence alignment of multiple Cas4 homologs. Residues predicted to coordinate the Fe-S cluster are boxed in orange and residues involved in catalytic metal ion binding boxed in pink. Cas4 sequences were derived from: Fno - *Francisella novicida*; Mla - *Moraxella lacunata*; Ain - *Acinetobacter indicus*; Tpe - *Treponema peruense*; Nar - *Nanoarchaeota archaeon*; Sli - *Saccharobesus litoralis*; Pdi - *Parabacteroides distasonis*; Cpa - *Candidatus Paceibacterota*; Xmu - *Xylanibacter muris*; Lil - *Leptospira ilyithenensis*. **(B)** Structure model of (Cas4)_1_– (Cas1)_4_–(Cas2)_2_ complex with a magnesium ion bound in the Cas4 active site. The Mg^2+^ is labelled in yellow, the residues coordinating the Fe-S cluster and Mg^2+^ are highlighted. Structure complex was predicted using the AlphaFold server (1).

# SUPPLEMENTARY TABLES

### Table S1. Bacterial strains used in this study.

| **Strain** | **Usage/ Genotype** | **Source** |
| --- | --- | --- |
| ***Escherichia coli*** | | |
| BL21(DE3) | T7-based *cas* gene expression for heterologous system/ B strain, *fhuA2 [lon] ompT gal (λ DE3) [dcm] ∆hsdS, λ DE3 = λ sBamHIo ∆EcoRI-B int::(lacI::PlacUV5::T7 gene1) i21 ∆nin5* | NEB |
| BL21(AI) | T7-based *cas* gene expression for heterologous system/ B strain, *F- ompT hsdSB (rB-mB-) gal dcm araB::T7RNAPtetA* | Invitrogen |
| NEB 5-alpha | Cloning strain/ K12 strain, *fhuA2 D(argF-lacZ)U169 phoA glnV44* Φ*80D(lacZ)M15 gyrA96 recA1 relA1 endA1 thi-1 hsdR17* | NEB |
| Rosetta 2(DE3) pLysS | T7-based *cas* gene expression for protein purification/ BL21 strain, *F- ompT hsdS*_B_(r_B_^-^ m_B_^-^) *gal dcm* (DE3) pLysSRARE2 (Cam^R^) | Novagen |
| INVαF' | Cloning of *in vitro* integration products/ *F´ endA1 recA1 hsdR17* (r_k_^-^, m_k_^+^) *supE44 thi-1 gyrA96 relA1* Φ*80lacZ∆M15 ∆(lacZYA-argF)U169 λ^-^* | Thermo |
| ***Francisella tularensis* subsp. *novicida*** | | |
| U112 | Amplification of *cas* genes/ wild type | David Weiss’ Lab |

### Table S2. Plasmids used in this study.

| **Plasmid** | **Characteristics ^a^** | **Source/ Reference** |
| --- | --- | --- |
| **General vectors** | | |
| pCDFDuet-1 (pEC574) | CloDF13 *ori*, P_T7_/MCS-1, P_T7_/MCS-2, P*_lacI_*/*lacI*, Sm^R^ | Novagen |
| pUC19 (pEC707) | ColE1 *ori*, P*_lac_*/*lacZα*, Amp^R^ | (2) |
| pEC-A-HI-SUMO (pEC1076) | f1 *ori*, ColE1 *ori*, *rop*, P*_lacI_*/*lacI*, P_T7_/H_6_-SUMO, Amp^R^ | Elena Conti’s Lab |
| pACYC184 (pEC2549) | p15A *ori*, Cam^R^, Tet^R^ | ATCC (3) |
| pCRII | ColE1 *ori*, P*_lac_*/*lacZα*, Neo^R^/Kan^R^, Amp^R^ | Thermo |
| **Cas protein over-expression** | | |
| pEC1862 | pEC-A-Hi-SUMOΩH_6_-sumo-*cas1* | This study |
| pEC2234 | pEC-A-Hi-SUMOΩH_6_-sumo-*cas1(*E242Q*)* | This study |
| pEC2236 | pCDFDuet-1ΩH_6_-*cas2* | This study |
| pEC2282 | pEC-A-Hi-SUMOΩH_6_-sumo-*cas1(*K120A*)* | This study |
| pEC2345 | pEC-A-Hi-SUMOΩH_6_-sumo-*cas4* | This study |
| pEC2436 | pCDFDuet-1ΩH_6_-*cas2(*D7N*)* | This study |
| pEC2474 | pCDFDuet-1 P_T7_/ΩH_6_-*cas4-* H_6_-*cas1-cas2)* | This study |
| ***in vitro* integration assay** | | |
| pEC2427 | pUC19Ω*CRISPR* | This study |
| **Heterologous system** | | |
| pEC2400 | pACYC184 P_T7_/Ω*-leader-CRISPR (sp1)* | This study |
| pEC2401 | pCDFDuet-1 P_T7_/Ω*(cas4-cas1-cas2)* | This study |
| pEC2411 | pCDFDuet-1 P_T7_/Ω*cas12a­* P_T7_/Ω*(cas4-cas1-cas2)* | This study |
| pEC2455 | pCDFDuet-1 P_T7_/Ω*cas12a* P_T7_/Ω*(cas4*(L6^STOP^)*-cas1-cas2)* | This study |
| pEC2457 | pCDFDuet-1 P_T7_/Ω*cas12a* P_T7_/Ω*(cas4*(D78N)*-cas1-cas2)* | This study |
| pEC2458 | pCDFDuet-1 P_T7_/Ω*cas12a* P_T7_/Ω*(cas4-cas1*(E242Q)*-cas2)* | This study |
| pEC2462 | pCDFDuet-1 P_T7_/Ω*cas12a* P_T7_/Ω*(cas4-cas1-cas2*(D7N)*)* | This study |
| pEC2472 | pCDFDuet-1 P_T7_/Ω*cas12a­*(E1006A) P_T7_/Ω*(cas4-cas1-cas2)* | This study |
| pEC2475 | pCDFDuet-1 P_T7_/Ω*cas12a­*(K671A) P_T7_/Ω*(cas4-cas1-cas2)* | This study |
| pEC2525 | pCDFDuet-1 P_T7_/Ω*(cas1-cas2)* | This study |
| pEC2664 | pCDFDuet-1 P_T7_/Ω*cas12a* P_T7_/Ω*(cas4-cas1-cas2*(D61A)*)* | This study |
| pEC2665 | pCDFDuet-1 P_T7_/Ω*cas12a* P_T7_/Ω*(cas4-cas1-cas2*(S9A)*)* | This study |
| pEC2666 | pACYC184 Ω*-leader-CRISPR (sp1)* | This study |
| pEC2713 | pCDFDuet-1 P_T7_/Ω*cas12a­* P_T7_/Ω*(cas1-cas2)* | This study |
| pEC3083 | pASK-IBA3plus-FliC | Marc Erhardt’s Lab |

**^a^ Abbreviations: H_6_ – 6x histidine-tag; MCS – multiple cloning site; P_x_ – promoter_name_; ^R^ – resistance, SUMO – small ubiquitin-like modifier protein tag; antibiotics: Amp – ampicillin, Cam – chloramphenicol, Kan – kanamycin, Neo – neomycin, Sm – streptomycin, Tet - tetracyclin.**

### Table S3. Oligonucleotides used in this study.

| **Oligo** | **5′ to 3′ sequence** | **F/R^b^** | **Purpose/ Target** |
| --- | --- | --- | --- |
| **Cloning** | | | |
| OLEC2445 | GATTTTGGATCCGATGTTGATAGTCAGTTATGATTTTAG | F | pEC2236 |
| OLEC2446 | TACTCGGCGGCCGCTTAGTCTATAAAAACAACCTCTTG | R | pEC2236 |
| OLEC6575 | GGTTTGGGTACCTTAGTCTATAAAAACAACCTCTTG | R | pEC2713 |
| OLEC6588 | ACCAGGAACAAACCGGCGGCCGCTCGATGTTTAGTAAAAATGATATT  GAATCAAAG | F | pEC1862 |
| OLEC6589 | GCAAAGCACCGGCCTCGTTATAACTGACTATCAACATACAC | R | pEC1862 |
| OLEC8895 | GCGGCCGCTCGATGGAAAATTATATTTTGATAACATGGTTAAATG  ATTTTATATTCTGTCC | F | pEC2345 |
| OLEC8896 | GGACAGAATATAAAATCATTTAACCATGTTATCAAAATATAATTTT  CCATCGAGCGGCCGC | R | pEC2345 |
| OLEC9221 | GATATACCATGGGCATGTCAATTTATCAAGAATTTGTTAATAAATATAG | F | pEC2411/ *cas12a* |
| OLEC9222 | GCGGCCGCATAATGTTAGTTATTCCTATTCTGCACGAAC | R | pEC2411/ *cas12a* |
| OLEC9223 | TTTTTTAAGCTTTAATACGACTCACTATAGGATGCGATTCATAGA  GAACAAGAGGTTG | F | pEC2400 |
| OLEC9224 | CGTAGAGGATCCCAAAAAACCCCTCAAGACCCGTTTAGAGGCCCC  AAGGGGTTATGCTAGGAATTATCTGAAGGCACAGGAATAGTAGC | R | pEC2400 |
| OLEC9230 | GATATACATATGGAAAATTATATTTTGATAACATGGTTAAATG | F | pEC2411/ pEC2401  *cas4-cas1-cas2* |
| OLEC9231 | GACGTCGGTACCTTAGTCTATAAAAACAACCTCTTGTTCTC | R | pEC2411/ pEC2401  *cas4-cas1-cas2* |
| OLEC9520 | ATGGGCAGCAGCCATCACCATCATCACCACAGCCAGGATCCGATG  GAAAATTATATTTTGATAACATGGTTAAATG | F | pEC2474 |
| OLEC9521 | CGGATCCTGGCTGTGGTGATGATGGTGATGGCTGCTGCCCATAT  GTATATCTCCTTCTTATACTTAACTAATATACTAAG | R | pEC2474 |
| OLEC10109 | AGATATGAATTCGATGTTTAGTAAAAATGATATTGAATCAAAGAA  TATAG | F | pEC2525 |
| OLEC10110 | CTCGAGGCGGCCGCTTAGTCTATAAAAACAACCTCTTGTTCTC | R | pEC2525 |
| OLEC10497 | AACTACTAAGTTCTTAGAGATATTTAAAAATATGACTGTTGTTATA  TATC | F | pEC2666 |
| OLEC10498 | CAACAGTCATATTTTTAAATATCTCTAAGAACTTAGTAGTTACTTA  TATCGTATGGGGCTGACTTCAGG | R | pEC2666 |
| OLEC10821 | GATATACATATGTTTAGTAAAAATGATATTGAATC | F | pEC2713 |
| **Site-directed mutagenesis ^a^** | | | |
| OLEC6446 | CTCTTTTAAATCCAAAATTTAAATCCGCAAAAACCACAATAGCATT  ATACTCTATAACT | F | pEC2472 |
| OLEC6447 | AGTTATAGAGTATAATGCTATTGTGGTTTTTGCGGATTTAAATTT  TGGATTTAAAAGAG | R | pEC2472 |
| OLEC6551 | AGATTTAGCAGAAAAGAAAACCGCAGGTAACATTTTATTTGCGCC  AGGTAA | F | pEC2475 |
| OLEC6552 | TTACCTGGCGCAAATAAAATGTTACCTGCGGTTTTCTTTTCTGCTA  AATCT | R | pEC2475 |
| OLEC8446 | GTAAATCCCTAGTTTGTGACTTTGTT**C**AGCCATTTAGATGTATAG | F | pEC2234 & pEC2458 |
| OLEC8447 | CTATACATCTAAATGGCT**G**AACAAAGTCACAAACTAGGGATTTAC | R | pEC2234 & pEC2458 |
| OLEC8604 | ATATAAGTTTAGCAAGGATTTTTATAACATCA**GC**GATACGCAATCAACAT  AACTTAGTCAAAAG | F | pEC2282 |
| OLEC8605 | CTTTTGACTAAGTTATGTTGATTGCGTATC**GC**TGATGTTATAAAAATCCT  TGCTAAACTTATAT | R | pEC2282 |
| OLEC9472 | GGATCCGATGTTGATAGTCAGTTAT**A**ATTTTAGTAATAATAAAGTACGTGC | F | pEC2436 |
| OLEC9473 | GCACGTACTTTATTATTACTAAAAT**T**ATAACTGACTATCAACATCGGATCC | R | pEC2436 |
| OLEC9474 | AAGTATAAGAAGGAGATATACATATGGAAAATTATATTTAGATAACATGGTTAAATGATTTTATATTC | F | pEC2455 |
| OLEC9475 | GAATATAAAATCATTTAACCATGTTATCTAAATATAATTTTCCATATGTATATCTCCTTCTTATACTT | R | pEC2455 |
| OLEC9478 | ACCATAAATATGGTTTGGTTGGTAAAATT**A**ATGTTTTTCATAAAGATAAG  GGC | F | pEC2457 |
| OLEC9479 | GCCCTTATCTTTATGAAAAACAT**T**AATTTTACCAACCAAACCATATTTATGGT | R | pEC2457 |
| OLEC9560 | AGGGTGTATGTTGATAGTCAGTTAT**A**ATTTTAGTAATAATAAAGTACGTGC | F | pEC2462 |
| OLEC9561 | GCACGTACTTTATTATTACTAAAAT**T**ATAACTGACTATCAACATACACCCT | R | pEC2462 |
| OLEC10253 | ATGTACCACTATTTACAAATGCTG**C**TAGTGTTTTAATCTTTAATGCTCC | F | pEC2664 |
| OLEC10254 | GGAGCATTAAAGATTAAAACACTA**G**CAGCATTTGTAAATAGTGGTACAT | R | pEC2664 |
| OLEC10507 | TGTATGTTGATAGTCAGTTATGATTTT**GC**TAATAATAAAGTACGTGCAAA  GTTTGC | F | pEC2665 |
| OLEC10508 | GCAAACTTTGCACGTACTTTATTATTA**GC**AAAATCATAACTGACTATCAA  CATACA | R | pEC2665 |
| **PCR-based acquisition screen** | | | |
| OLEC3771 | CAGTGGCCTTATTAAATGACTTC | R | *in vivo* & *in vitro* acquisition screen |
| OLEC6679 | GAGATATTTAAAAATATGACTGTTG | F | *in vivo* acquisition screen |
| OLEC10195 | CGGTACCGAGGTCTTCC | F/R | *in vitro* acquisition screen |
| OLEC10196 | CCTCGGTACCGTTATCTTG | F/R | *in vitro* acquisition screen |
| OLEC10197 | CTTTTTTTGCTGATTTAGGCAAAAAC | F | *in vitro* acquisition screen |
| OLEC14483 | TAGGCAAAAACGGGTCTA | F | *In vivo* spacer acquisition library |
| OLEC14484 | TATTCTTTCCCCTGCACTGT | R | *In vivo* spacer acquisition library |
| OLEC14485 | TTCTTTCCCCTGCACTGTNNNNNNNNNNATTAAATGACTTCTCATCTA | R | *In vivo* spacer acquisition library |
| OLEC14486 | AATGATACGGCGACCACCGAGATCTACACTCTTTCCCTACACGACGCTCTTCCGATCTTAGGCAAAAACGGGTCTA | F | *In vivo* spacer acquisition library |
| OLEC11357-11386^c^ | CAAGCAGAAGACGGCATACGAGATNNNNNNNNGTGACTGGAGTTCAGACGTGTGCTCTTCCGATCTTCTACTATTCTTTCCCCTGCACTGT | R | *In vivo* spacer acquisition library |
| ***in vitro* pre-spacer substrates** | | | |
| OLEC7123 | TCGCAAGATAACGGTACCGAGGTCTTCCAG | F | annealed to 7124 or  single-stranded |
| OLEC7124 | CTGGAAGACCTCGGTACCGTTATCTTGCGA | R | annealed to 7123 |
| OLEC7283 | AGATAACGGTACCGAGGTCTTCCAG | F | annealed to 7944 or  annealed to 8961 |
| OLEC7944 | AGACCTCGGTACCGTTATCTTGCGA | R | annealed to 7283 |
| OLEC8831 | CAAGATAACGGTACCGAGGTCTTCCAG | F | annealed to 8959 |
| OLEC8959 | TGGAAGACCTCGGTACCGTTATCTTGC | R | annealed to 8831 |
| OLEC8833 | AAGATAACGGTACCGAGGTCTTCCAG | F | annealed to 8960 |
| OLEC8960 | GGAAGACCTCGGTACCGTTATCTTGC | R | annealed to 8833 |
| OLEC8961 | GAAGACCTCGGTACCGTTATCTTGC | R | annealed to 7283 |
| OLEC8835 | GATAACGGTACCGAGGTCTTCCAG | F | annealed to 8962 |
| OLEC8962 | AAGACCTCGGTACCGTTATCTTGC | R | annealed to 8835 |
| OLEC8837 | ATAACGGTACCGAGGTCTTCCAG | F | annealed to 8908 |
| OLEC8908 | AGACCTCGGTACCGTTATCTTGC | R | annealed to 8837 |
| OLEC8839 | TAACGGTACCGAGGTCTTCCAG | F | annealed to 8936 |
| OLEC8963 | GACCTCGGTACCGTTATCTTGC | R | annealed to8839 |
| OLEC8964 | AACGGTACCGAGGTCTTCCAG | F | annealed to 9865 |
| OLEC9865 | ACCTCGGTACCGTTATCTTGC | R | annealed to 8964 |
| OLEC9013 | ATAAGGTACCGGTCTTCCAG | F | annealed to 9014 |
| OLEC9014 | AGACCGGTACCTTATCTTGC | R | annealed to 9015 |
| OLEC9015 | ATAAGGTACCAGGTCTTCCAG | F | annealed to 9016 |
| OLEC9016 | AGACCTGGTACCTTATCTTGC | R | annealed to 9015 |
| OLEC9017 | ATAAGGTACCGAGGTCTTCCAG | F | annealed to 9018 |
| OLEC9018 | AGACCTCGGTACCTTATCTTGC | R | annealed to 9017 |
| OLEC9019 | ATAACTGGTACCGAGGTCTTCCAG | F | annealed to 9020 |
| OLEC9020 | AGACCTCGGTACCAGTTATCTTGC | R | annealed to 9019 |
| OLEC9021 | AGATAACAGGTACCGAGGTCTTCCAG | F | annealed to 9022 |
| OLEC9022 | AGACCTCGGTACCTGTTATCTTGCGA | R | annealed to 9021 |
| OLEC9023 | AGATAACATGGTACCGAGGTCTTCCAG | F | annealed to 9023 |
| OLEC9024 | AGACCTCGGTACCATGTTATCTTGCGA | R | annealed to 9024 |
| OLEC10237 | ATAACGGTACCGAGGTCTTCCA[ddC] | F | annealed to 10238 |
| OLEC10238 | AGACCTCGGTACCGTTATCTTG[ddC] | R | annealed to 10239 |
| ***in vitro* CRISPR substrates (hairpins)^a^** | | | |
| OLEC7532 | GCAAAAACGGGTCTAAGAACTTTAAATAATTTCTACTGTTGTAGATGAGA  AGTCATAAACCCATGACTTCTCAT | F | wt CRISPR |
| OLEC8983 | GCAAAAACGG**CAGATT**GAACTTTAAATAATTTCTACTGTTGTAGATGAGA  AGTCATAAACCCATGACTTCTCAT | F | repeat mutant  (1 to 6) |
| OLEC8984 | GCAAAAACGGGTCTAA**CTTGAA**TAAATAATTTCTACTGTTGTAGATGAGA  AGTCATAAACCCATGACTTCTCAT | F | repeat mutant  (7 to 12) |
| OLEC8985 | GCAAAAACGGGTCTAAGAACTT**ATTTAT**ATTTCTACTGTTGTAGATGAGA  AGTCATAAACCCATGACTTCTCAT | F | repeat mutant  (13 to 18) |
| OLEC8986 | GCAAAAACGGGTCTAAGAACTTTAAATA**TAAAGA**ACTGTTGTAGATGAGA  AGTCATAAACCCATGACTTCTCAT | F | repeat mutant  (19 to 24) |
| OLEC8987 | GCAAAAACGGGTCTAAGAACTTTAAATAATTTCT**TGACAA**GTAGATGAGA  AGTCATAAACCCATGACTTCTCAT | F | repeat mutant  (25 to 30) |
| OLEC8988 | GCAAAAACGGGTCTAAGAACTTTAAATAATTTCTACTGTT**CATCTA**GAGA  AGTCATAAACCCATGACTTCTCTA | F | repeat mutant  (31 to 36) |
| OLEC8989 | GCAAAAA**GCC**GTCTAAGAACTTTAAATAATTTCTACTGTTGTAGATGAGA  AGTCATAAACCCATGACTTCTCAT | F | leader mutant  (-3 to -1) |
| OLEC8990 | GCAA**TTT**CGGGTCTAAGAACTTTAAATAATTTCTACTGTTGTAGATGAGA  AGTCATAAACCCATGACTTCTCAT | F | leader mutant  (-6 to -4) |
| OLEC8991 | G**GTT**AAACGGGTCTAAGAACTTTAAATAATTTCTACTGTTGTAGATGAGA  AGTCATAAACCCATGACTTCTCAT | F | leader mutant  (-9 to -7) |
| OLEC9186 | GCAAAAACGGGTCTAAGAACTTTAAATATTTCTACTGTTGTAGATGAGA  AGTCATAAACCCATGACTTCTCAT | F | repeat mutant (-1) |
| OLEC9187 | GCAAAAACGGGTCTAAGAACTTTAAATTTTCTACTGTTGTAGATGAGAA  GTCATAAACCCATGACTTCTCAT | F | repeat mutant (-2) |
| OLEC9188 | GCAAAAACGGGTCTAAGAACTTTAAATTTCTACTGTTGTAGATGAGAAG  TCATAAACCCATGACTTCTCA | F | repeat mutant (-3) |
| OLEC9189 | GCAAAAACGGGTCTAAGAACTTTAAATAA**A**TTTCTACTGTTGTAGATGA  GAAGTCATAAACCCATGACTTCTCAT | F | repeat mutant (+1) |
| OLEC9190 | GCAAAAACGGGTCTAAGAACTTTAAATAA**TA**TTTCTACTGTTGTAGATGA  GAAGTCATAAACCCATGACTTCTCAT | F | repeat mutant (+2) |
| OLEC9191 | GCAAAAACGGGTCTAAGAACTTTAAATAA**TAA**TTTCTACTGTTGTAGATG  AGAAGTCATAAACCCATGACTTCTCAT | F | repeat mutant (+3) |
| ***In vitro* Cas4 enzymatic assay** | | | |
| OLEC9419 | AAAAAAAAAAGCAAGATAACTGGTACCGAGGTCTTCCAGAAAAAAAAAA | F | ssDNA target |
| OLEC9900 | GCAAGATAACTGGTACCGAGGTCTAGTCCCCCCCCCC | F | annealed to 9901 |
| OLEC9901 | ACTAGACCTCGGTACCAGTTATCTTGCCCCCCCCCC | R | annealed to 9902 |
| OLEC9908 | GCAAGATAACTGGTACCGAGGTCTAGTCTTTCCCCCC | F | annealed to 9909 |
| OLEC9909 | ACTAGACCTCGGTACCAGTTATCTTGCGTTTCCCCCC | R | annealed to 9908 |
| OLEC9910 | GCAAGATAACTGGTACCGAGGTCTAGTCAAACCCCCC | F | annealed to 9911 or ssDNA target |
| OLEC9911 | ACTAGACCTCGGTACCAGTTATCTTGCGAAACCCCCC | R | annealed to 9910 |
| OLEC14866 | CAACAAGATAGAATTACCTTTTAATCTTAAATTATTATATCCAGAAACTATTGATGGTAC | F | ssDNA target, 5′ FAM labelled |

**^a^ Bold letter indicate mutated nucleotides.**

**^b^ F/R, Forward/Reverse.**

**^c^ The NNNNNNNN is standard P7 barcode (index) sequences for Illumina sequencing**

### Table S4. Hot spots on *E. coli* BL21 genome for spacer acquisition.

| **Location** | **Counts ^a^** | **Protospacer**  **Gene** |
| --- | --- | --- |
| 26729 | 195 | CCGTGGCAGTCCCAGCCAGGCAAAAACGG  ileS, isoleucyl-tRNA synthetase |
| 335249 | 21 | GGTGATGGTGCTGCGCTGGAGTGACGGCAGTTAT  lacZ, beta-D-galactosidase |
| 624123 | 79 | GGACATCTGCGTTCTGTTCGGCGAGCA  dacA, D-alanyl-D-alanine carboxypeptidase (penicillin-binding protein 5) |
| 731601 | 21 | GCTGCTTGGGCCGAGGCGTGCAAACT  cydA, cytochrome d terminal oxidase, subunit I |
| 739850 | 22 | CGAGCGAAGCGAGTCATCCTGCACGAG  non-coding |
| 814177 | 20 | GCAATCAGGCGTGTCAGGTGGCTGTGTAG  ybiH, DUF1956 domain-containing tetR family putative transcriptional regulator |
| 1447848 | 91 | GTTTTCCCGGCCGTTGCCACCTCATCCGA  trg, methyl-accepting chemotaxis protein III, ribose and galactose sensor receptor |
| 1533395 | 28 | CAGGTTGGTCTTGGGAAATTGGCTGCGCGT  yddA, putative multidrug ABC transporter permease/ATPase |
| 1540827 | 30 | CTGTACGTTCTGGGTACCATGTTCGTGCT  ydeP, putative oxidoreductase |
| 1567280 | 25 | CGTATTACGCCGGGCAACTGGTGATGGAT  marC, UPF0056 family inner membrane protein |
| 1844548 | 30 | ACTTTTACGCCTGACGATCGACCGTGCCCTGA  yoaD, putative membrane-anchored cyclic-di-GMP phosphodiesterase |
| 1856670 | 114 | GTTTCGCTCAGGGGCAATCGCTGACGTT  yebQ, putative transporter |
| 2412519 | 20 | ACAAAACGGGTCGCTGGTTCGCGCCATACCTG  cysA, sulfate/thiosulfate transporter subunit |
| 2455317 | 229 | CCTGGGTTATCGGCGGGGCGGCGATTTT  uraA, uracil permease |
| 2564601 | 30 | CTCCGCTGCGGCTGTACAGTGGCTATAG  pgpC, phosphatidylglycerophosphatase C, membrane bound |
| 2667879 | 70 | CCTTTAGGCCCGGCAACCACGCCGTTGGTT  flhA, formate hydrogenlyase transcriptional activator FlhA |
| 3201526 | 83 | ACGGATTCGCCACCGAAACGCTTAACG  rpmA, 50S ribosomal subunit protein L27 |
| 3475336 | 29 | AAACGTTGGCCCTGATATGTATCGGATAGT  acpT, 4'-phosphopantetheinyl transferase |
| 3513323 | 29 | AATAATTTGTTGTGTAAACCACTGATAA  non-coding |
| 3632716 | 104 | TGGTGATGGTAGACGACTCCCACGCGGTCGG  kbl, glycine C-acetyltransferase |
| 3645773 | 22 | GTCACCTTTCCAGCCAGCGCCGTTACTG  rfaQ, lipopolysaccharide core heptosyltransferase RfaQ |
| 3713282 | 22 | GGGACTGAGGAGGCCGAACGTCAGAACGG  ECD03522, Single-strand binding protein (SSB) (Helix-destabilizing protein) |
| 3774398 | 64 | TCTCGCCAGCGTTCTGCACGTTGTAGTTG  yidC, membrane protein insertase |
| 3816239 | 50 | CTCTCCCGCAAGGTTCCGCAGCTGTGTAAA  ilvD,dihydroxy-acid dehydratase |
| 3833019 | 23 | GSU80_18495  UDP-N-acetylglucosamine 2-epimerase (non-hydrolyzing) |
| 4141419 | 34 | CACGTAGCGCTGCGTAACCGTAGCAATAC  pgi, glucosephosphate isomerase |
| 4170292 | 23 | GGTCACCGGCGTTTCGTTGCTGGGCATTAT  pspG, phage shock protein G |
| 4327250 | 45 | TCGGTATCGACACCGTGCAGGCGATGGCAGG  ulaA, L-ascorbate-specific enzyme IIC permease component of PTS |
| 4529096 | 28 | CAAACTGCAGCACACCAACTGCACCAACGAT  prfC, peptide chain release factor RF-3 |
| 2277047/  2519142 | 160 **^b^** | CGAGTTGCGCATGGCTATCGATAATTAC  ECD_02200, YadA/ ECD_04289, transposase |
| 1257504  1258574  1258039 | 30 **^b^** | GTTTTACCTCTCAACGTGCGGGGGTTTTCT  rdlA/B/C, sRNA antisense regulator affects LdrA/B/C translation; proposed addiction module in LDR-A repeat, with toxic peptide LdrA/B/C |

**^a^ Most frequently acquired spacers with counts over 20 in single sample.**

**^b^ Protospacers mapped to multiple positions on genome.**

# REFERENCES

1. Abramson,J., Adler,J., Dunger,J., Evans,R., Green,T., Pritzel,A., Ronneberger,O., Willmore,L., Ballard,A.J., Bambrick,J., *et al.* (2024) Accurate structure prediction of biomolecular interactions with AlphaFold 3. *Nature*, **630**, 493–500.

2. Yanisch-Perron,C., Vieira,J. and Messing,J. (1985) Improved M13 phage cloning vectors and host strains: nucleotide sequences of the M13mp18 and pUC19 vectors. *Gene*, **33**, 103–119.

3. Chang,C.-H., Chang,H.-Y., Rappsilber,J. and Ishihama,Y. (2021) Isolation of Acetylated and Unmodified Protein N-Terminal Peptides by Strong Cation Exchange Chromatographic Separation of TrypN-Digested Peptides. *Molecular & Cellular Proteomics*, **20**, 100003.
